# Unique and shared inflammatory profiles of human brain endothelia and pericytes

**DOI:** 10.1186/s12974-018-1167-8

**Published:** 2018-05-11

**Authors:** Leon C. D. Smyth, Justin Rustenhoven, Thomas I.-H. Park, Patrick Schweder, Deidre Jansson, Peter A. Heppner, Simon J. O’Carroll, Edward W. Mee, Richard L. M. Faull, Maurice Curtis, Mike Dragunow

**Affiliations:** 1 Department of Pharmacology and Clinical Pharmacology, The University of Auckland, Private Bag 92019, Auckland, 1142 New Zealand; 2 Centre for Brain Research, The University of Auckland, Private Bag 92019, Auckland, 1142 New Zealand; 3 Department of Anatomy and Medical Imaging, The University of Auckland, Private Bag 92019, Auckland, 1142 New Zealand; 4 Auckland City Hospital, Auckland, 1023 New Zealand

**Keywords:** Neurovascular, Blood-brain barrier, Neuroinflammation, IL-1β, SMAD2/3, IGFBP, GM-CSF

## Abstract

**Background:**

Pericytes and endothelial cells are critical cellular components of the blood-brain barrier (BBB) and play an important role in neuroinflammation. To date, the majority of inflammation-related studies in endothelia and pericytes have been carried out using immortalised cell lines or non-human-derived cells. Whether these are representative of primary human cells is unclear and systematic comparisons of the inflammatory responses of primary human brain-derived pericytes and endothelia has yet to be performed.

**Methods:**

To study the effects of neuroinflammation at the BBB, primary brain endothelial cells and pericytes were isolated from human biopsy tissue. Culture purity was examined using qPCR and immunocytochemistry. Electrical cell-substrate impedance sensing (ECIS) was used to determine the barrier properties of endothelial and pericyte cultures. Using immunocytochemistry, cytometric bead array, and ECIS, we compared the responses of endothelia and pericytes to a panel of inflammatory stimuli (IL-1β, TNFα, LPS, IFN-γ, TGF-β_1_, IL-6, and IL-4). Secretome analysis was performed to identify unique secretions of endothelia and pericytes in response to IL-1β.

**Results:**

Endothelial cells were pure, moderately proliferative, retained the expression of BBB-related junctional proteins and transporters, and generated robust TEER. Both endothelia and pericytes have the same pattern of transcription factor activation in response to inflammatory stimuli but respond differently at the secretion level. Secretome analysis confirmed that endothelia and pericytes have overlapping but distinct secretome profiles in response to IL-1β. We identified several cell-type specific responses, including G-CSF and GM-CSF (endothelial-specific), and IGFBP2 and IGFBP3 (pericyte-specific). Finally, we demonstrated that direct addition of IL-1β, TNFα, LPS, and IL-4 contributed to the loss of endothelial barrier integrity in vitro.

**Conclusions:**

Here, we identify important cell-type differences in the inflammatory response of brain pericytes and endothelia and provide, for the first time, a comprehensive profile of the secretions of primary human brain endothelia and pericytes which has implications for understanding how inflammation affects the cerebrovasculature.

**Electronic supplementary material:**

The online version of this article (10.1186/s12974-018-1167-8) contains supplementary material, which is available to authorized users.

## Background

Blood vessels in the brain have a unique structure, being formed by specialised endothelia that have extensive contacts with pericytes, astrocyte endfeet, microglia, and neurons [1, 2]. Unlike blood vessels in most organs, cerebral blood vessels do not allow the leakage of plasma proteins and entry of immune cells from the blood into the parenchyma under normal circumstances, a property known as the blood-brain barrier (BBB) [3]. The BBB is primarily formed by a continuous layer of non-fenestrated endothelia linked by tight junctions [4–7], but is also regulated by pericytes [8, 9], which demonstrate an exceptionally high coverage of vessels in the brain [9, 10]. The maintenance of a functional BBB is essential to the regulation of the microenvironment in the brain parenchyma and prevents the exposure of neurons to potentially toxic blood-borne molecules like fibrinogen and haemoglobin [11, 12].

Inflammation is an important process that allows the removal of pathogens and toxins; however, inflammation can be particularly damaging to the brain. The brain is fully encased by the skull, meaning that even a small degree of swelling due to inflammation can lead to damaging increases in intracranial pressure. At the cellular level, the brain possesses a limited regenerative capacity and loss of neurons as a result of unchecked inflammation cannot be fully compensated through endogenous cellular replacement [13]. Neuronal death can be caused directly by inflammatory cytokines, or through the inflammatory activation of the brain’s resident immune cells, microglia [14, 15]. It is now understood that both BBB breakdown and inflammation are widespread in neurological diseases and are hallmarks of Alzheimer’s disease [16, 17], epilepsy [18, 19], stroke [20, 21], and multiple sclerosis [22–25].

In the brain, microglia and astrocytes are the most commonly studied neuroinflammatory cell types; however, blood vessels are another important site of cerebral inflammation [26]. The vasculature is perhaps a more significant target for inflammation in the brain than in other organs, due to the presence of the BBB [27]. The BBB has been shown to break down in inflammatory conditions [28, 29] and diseased states [12, 17, 23, 30–32] allowing entry of peripheral immune cells or toxic/inflammatory plasma products into the parenchyma [11]. Pericytes and endothelia therefore occupy a unique position, at the interface of the brain and the periphery, and may contribute to the propagation of systemic inflammation to the brain [29]. Conversely, vessels can be a target of neuroinflammation that has begun in the brain, with the potential to exacerbate dysfunction through opening of the BBB [33]. Brain endothelia are known to be sensitive to inflammation, secreting chemokines such as monocyte chemoattractant protein-1 (MCP-1), which draw circulating immune cells to an area of injury [34]. Inflammatory signalling in endothelia also induces the expression of cell adhesion molecules, making the vessel ‘sticky’ to immune cells and allowing for extravasation of peripheral immune cells into the brain parenchyma [35]. Recently, pericytes have garnered attention as mediators of inflammation in the brain, also secreting chemokines and expressing cell adhesion molecules [1, 36–38]. Interestingly, pericytes may play a unique role in immune infiltration, being able to ‘instruct’ immune cells following extravasation [39], assist in leukocyte crawling in the vessel [40], and alter their morphology to facilitate neutrophil extravasation [41]. Furthermore, there is mounting evidence that pericyte secretions may alter endothelial barrier function following inflammatory activation [42], as demonstrated in models of stroke [43]. Indeed, vascular clustering and pericyte reactivity is observed in diseases with an inflammatory component, such as stroke and epilepsy [18, 20], with migration of pericytes away from the vascular wall [19, 21], and may have implications for tissue fibrosis [44, 45].

Human brain biopsy tissue is a valuable resource for the study of neuroinflammation and aspects of human disease in pericytes and endothelia of the BBB [46, 47]. The growth of pure endothelia and pericyte cultures allows their inflammatory responses to be interrogated in isolation, providing cell-specific information. We demonstrate that endothelia retain BBB properties in vitro, and that barrier function is regulated strongly by inflammatory stimuli. Furthermore, we identify both distinct and overlapping immune responses between primary brain endothelia and pericytes that are important to understanding how inflammation may affect the BBB.

## Methods

### Tissue source

The experiments conducted here were approved by the Northern Regional Ethics Committee (New Zealand) for biopsy tissue and The University of Auckland Human Participants Ethics Committee for post-mortem brain tissue. All methods were carried out in accordance with the approved guidelines. Biopsy human brain tissue was obtained with informed written consent from the patient and family members (Additional file 1). Tissue used in this study was derived from paediatric epilepsy surgery, surgery for adult drug-refractive epilepsy, and normal brain regions resected from patients with deep tumours (Additional file 1: Table S1). This technique provided sufficient endothelial yields for experiments from as little as 0.25 g tissue. Cultures were also attempted from post-mortem brains (Additional file 1: Table S1), however yielded too few viable endothelia for experimentation.

### Tissue dissociation

The endothelial cell culture protocol was adapted to work in concert with a previously established method to generate mixed glial cultures from surgical specimens, routinely used in our laboratory [48]. Cultures of endothelial cells were attempted using autopsy specimens as well, but yielded few or no viable endothelial cells. Following collection of tissue, HBSS and meninges were removed. Tissue was then thoroughly mechanically dissociated with a sterile scalpel and enzymatically dissociated (10 mL/g tissue; 10 U/mL DNase I (Invitrogen, CA, USA), 2.5 U/mL papain (Worthington, NJ, USA) in Hibernate-A medium (Gibco, CA, USA)) for 15 min at 37 °C with agitation. The suspension was triturated, and then left to dissociate for a further 15 min at 37 °C with agitation. Digestion was quenched with the addition of one volume of media, either *complete DMEM* as per [48] (Dulbecco’s modified Eagle’s media with F12 supplement (DMEM:F12; Gibco) containing 10% FBS (Gibco, CA, USA), 1% penicillin/streptomycin (Gibco), and 1% GlutaMAX (Gibco)); *neural stem cell medium* as per [49, 50] (DMEM:F12 with 1% B27 supplement (Gibco), 1% penicillin/streptomycin, 1% GlutaMAX); or *neuronal medium* as per Park et al. (*In submission*) (DMEM:F12 with 1% vitamin A-containing B27 supplement (Gibco), 1% penicillin/streptomycin (Gibco), 1% GlutaMAX (Gibco), and BDNF, NGF, NT3, GDNF, and IGF-1 (Peprotech, NJ, USA) at 40 ng/mL). At this point, the suspension was allowed to settle and the supernatant, containing cell suspension, was passed through a 70- or 100-μm nylon strainer (Bector Dickinson, NJ, USA). The remaining debris and vessel component was triturated and passed through the strainer.

### Mixed glial and pericyte cultures

**Fig. 1:**
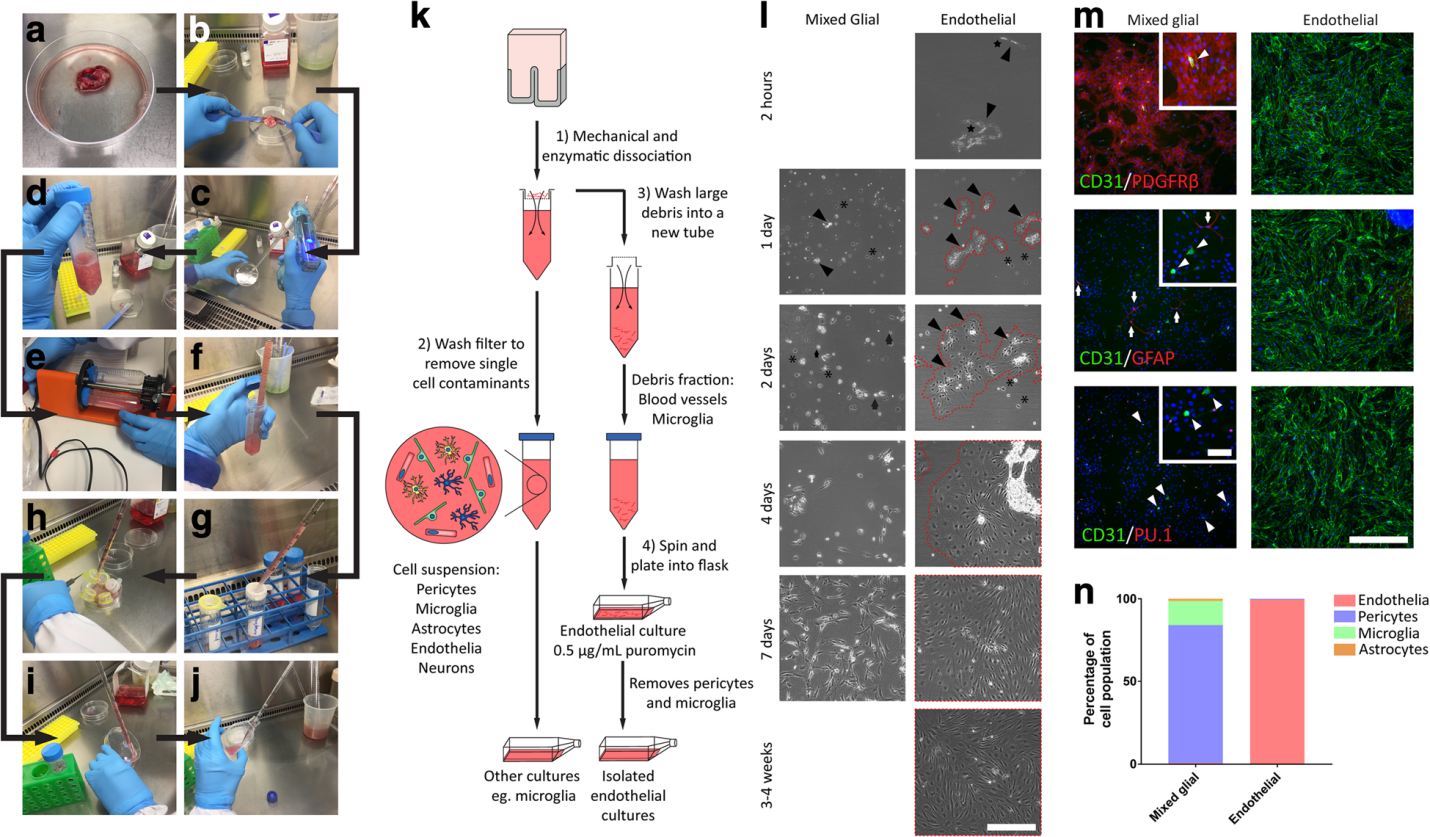
Generation of pure endothelial cultures from human brain tissue. Brain tissue was cultured to generate mixed glial and endothelial cultures. Cells were fixed, and purity of cultures was assessed at passage two using immunocytochemistry. **a** Processing of biopsy specimens, followed by **b** mechanical and **c**–**e** enzymatic digestion at 37 °C. Suspension was **f** triturated and **g** passed through cell strainers **h** which were washed. **i** Debris were collected, spun, and **j** plated onto a matrigel-coated flask. **k** Schematic of the method used to generate mixed glial (containing astrocytes/microglia/endothelia/pericytes), and isolated endothelial cultures. **l** Representative live images of passage 1 cultures throughout different growth stages. Arrowheads highlight blood vessels and endothelia while areas outlined in red are endothelial colonies, arrows indicate astrocytes and asterisks denote microglia. Scale bar = 200 μm. **m** Representative images and **n** quantification of staining for lineage markers (CD31, endothelial; PDGFRβ, pericyte; GFAP, astrocyte; PU.1, microglial) in passage two mixed glial and endothelial cultures. Scale bar = 500 μm, inset scale bar = 100 μm. **d** Cell lineage assessment of endothelial and mixed glial cultures (mean, *n* = 3 mixed glial, *n* = 5 endothelial)

**Fig. 2:**
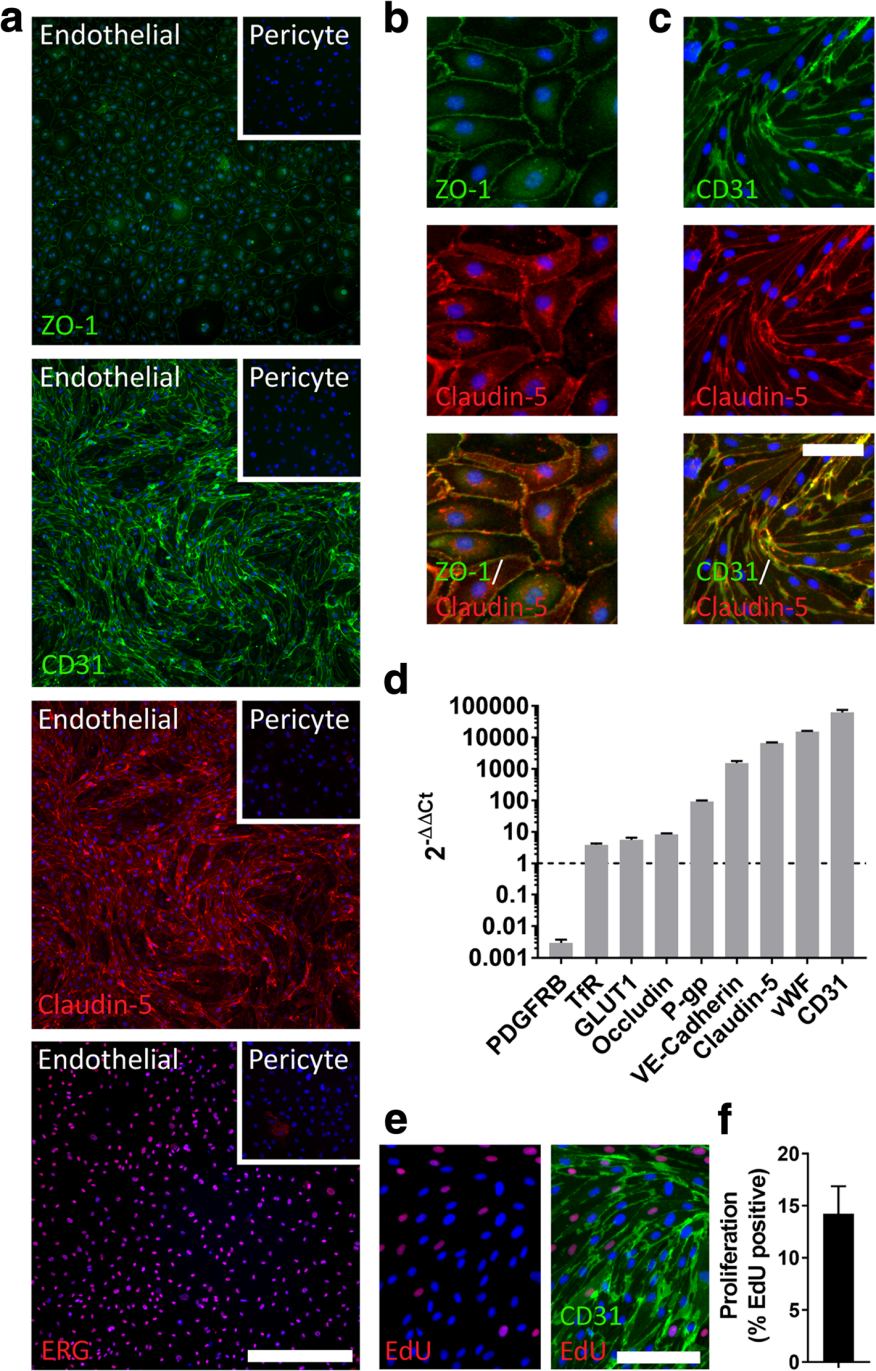
Isolated endothelia are proliferative and re-establish blood-brain barrier phenotype. Endothelial and pure pericyte cultures were treated with EdU (48 h, 10 μM) then fixed and stained for the junctional proteins ZO-1, claudin-5, CD31, endothelial nuclear marker ERG, and proliferation marker EdU. RNA was extracted from endothelia, and a single pure pericyte culture, and gene expression analysed by qPCR. **a** Representative images of immunocytochemical staining of zona occludens-1 (ZO-1), CD31, claudin-5, and ERG in endothelia and pericytes. Scale bar = 500 um. **b** High-magnification representative images of co-staining of CD31 and claudin-5, and **c** CD31 and claudin-5. Scale bar = 25 μm. **d** Gene expression analysis of endothelial cultures (mean ± SEM, *n* = 3), normalised to gene expression of a single pure pericyte culture. **e** Representative images of EdU staining in endothelia and **f** quantification of basal proliferation over 48 h

**Fig. 3:**
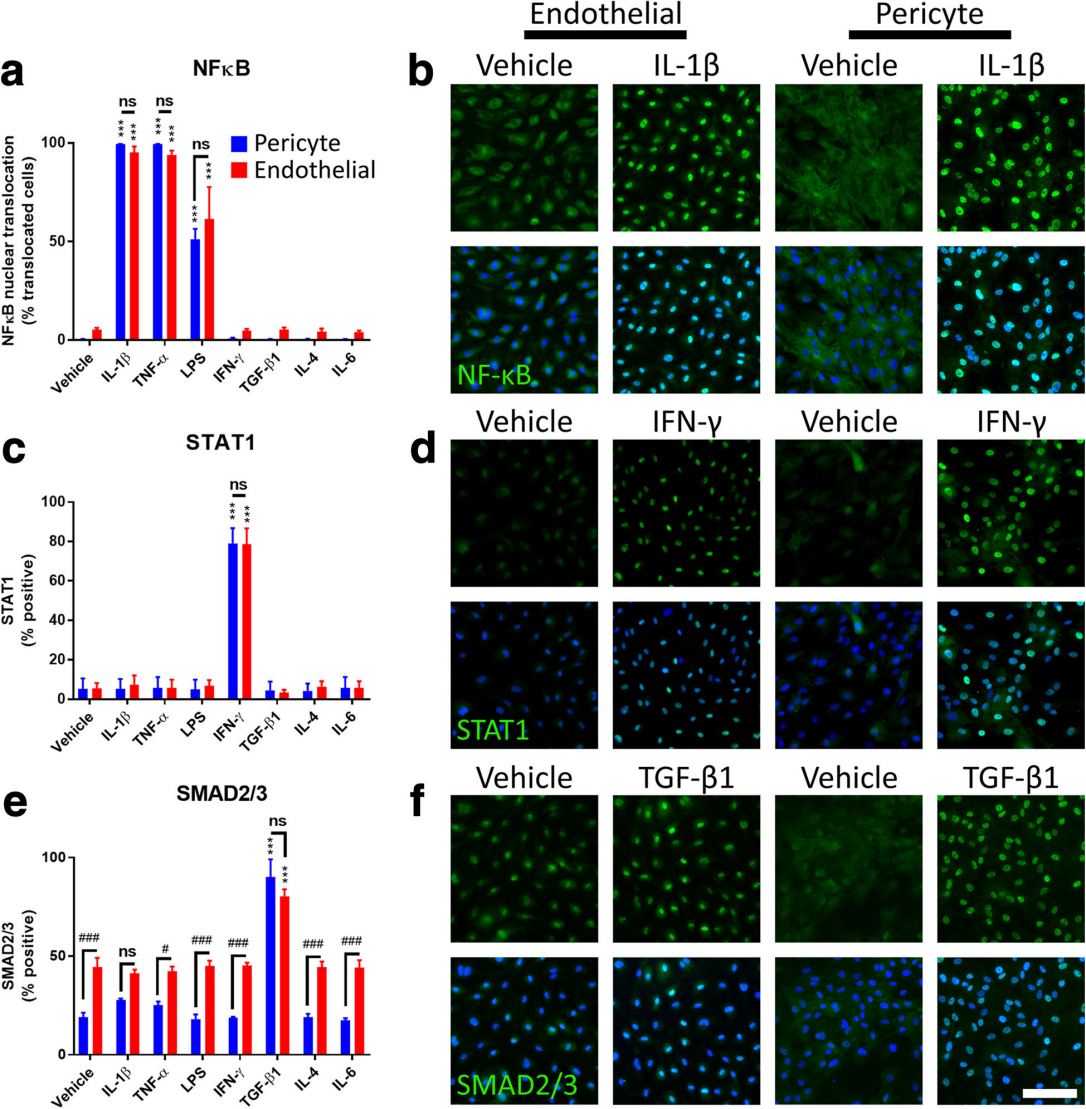
Inflammatory responses through the NF-κB, STAT1, and SMAD2/3 pathways are conserved between endothelia and pericytes. Endothelia and pericytes were cultured, before treatment with inflammatory mediators (10 ng/mL, 1 h) and transcription factor activation analysis by immunocytochemistry. **a** Quantification and **b** representative images of NF-κB translocation in endothelial (red) and pericyte (blue) cultures in response to inflammatory mediators assessed. **c** Quantification and **d** representative images of STAT1 activation in endothelial and pericyte cultures in response to inflammatory mediators assessed. **e** Quantification and **f** representative images of SMAD2/3 activation in endothelial and pericyte cultures in response to inflammatory mediators assessed. Scale bar = 200 μm. (*n* = 3, mean ± SEM)

**Fig. 4:**
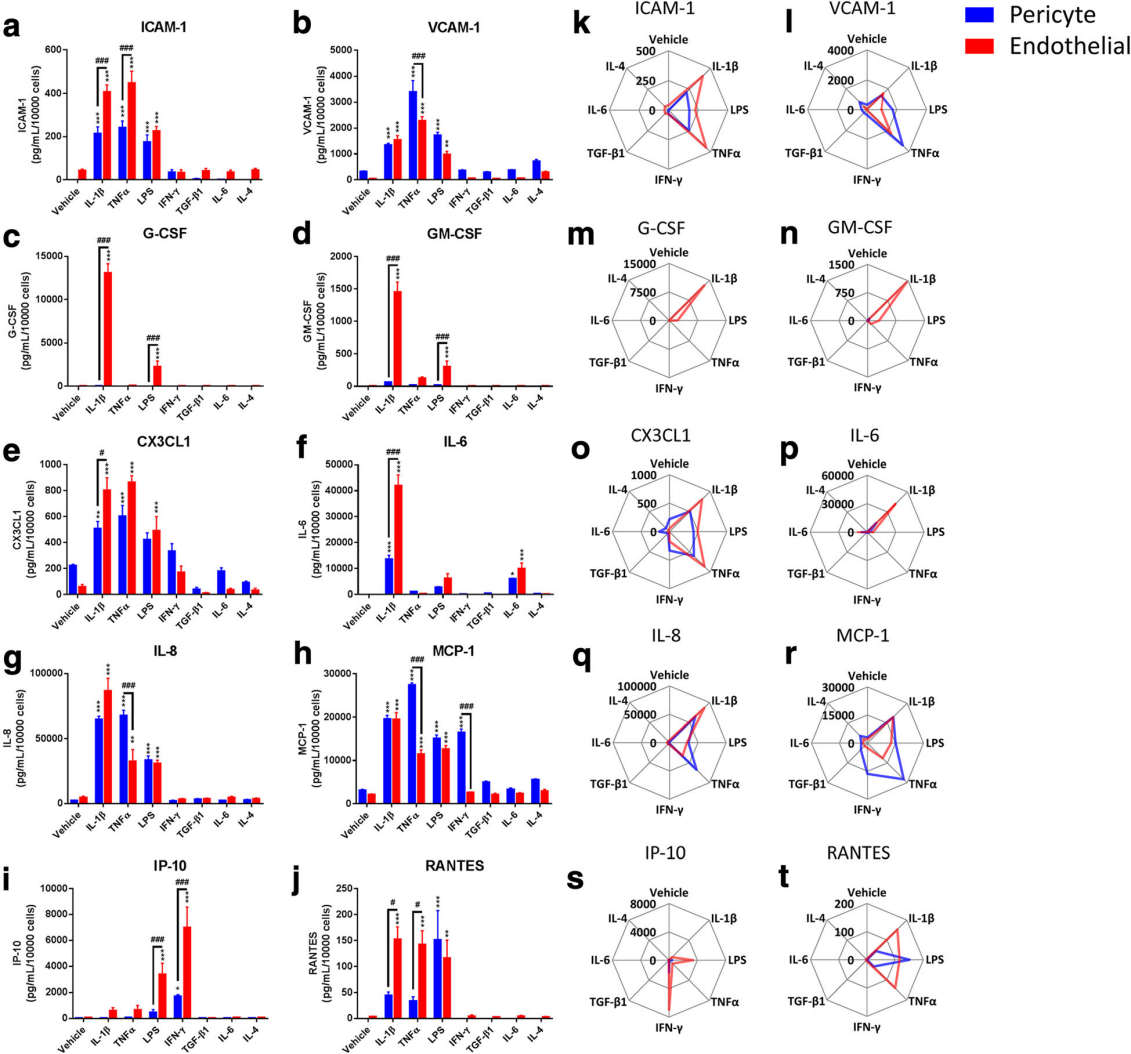
Endothelia and pericytes have unique responses to inflammatory stimuli. Endothelia and pericytes were cultured, and treated with inflammatory mediators (10 ng/mL, 24 h) before conditioned media were taken, and cytokine secretion analysed by cytometric bead array. Secretion of **a** ICAM-1, **b** VCAM-1, **c** G-CSF, **d** GM-CSF, **e** CX3CL1, **f** IL-6, **g** IL-8, **h** MCP-1, **i** IP-10, and **j** RANTES in response to inflammatory stimuli (*n* = 3–4, mean ± SEM). **k**–**t** Cytokine secretion data represented in radar charts (*n* = 3–4, mean)

**Fig. 5:**
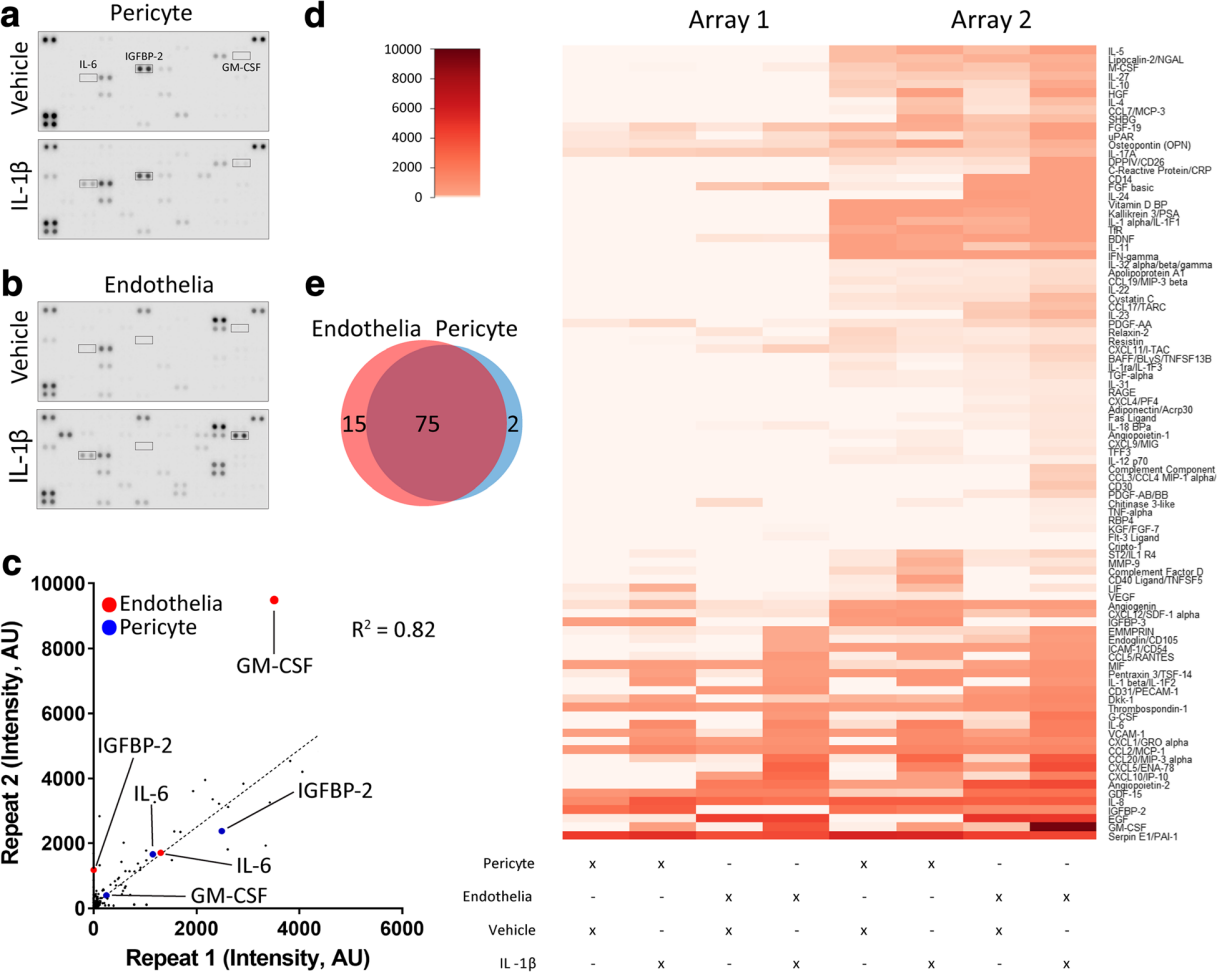
Brain pericytes and endothelia have overlapping but distinct responses to IL-1β. Endothelia and pericytes were cultured and treated with IL-1β (10 ng/mL, 24 h) or vehicle before conditioned media were taken, and cytokine secretion measured using a Proteome Profiler™ Human XL Cytokine Array Kit. Representative cytokine array blots of conditioned media from **a** pericytes and **b** endothelia treated with either vehicle or IL-1β. **c** Scatterplot of the correlation between spot intensities from each repeat. **d** Non-biased clustering analysis of secretions in endothelia and pericytes (*n* = 2 cases), and **e** Venn diagram of secretion profile of endothelia and pericytes

Components of the suspension that passed through the strainer contain other cerebral cell types and can be cultured to obtain pericytes [48], microglia [49], neural stem cells [50], and neurons (Park et al., *In submission*). Here, the single cell suspension was plated into a T75 and cultured in complete DMEM and left to generate a mixed glial culture containing pericytes, astrocytes, microglia, and endothelia and otherwise treated as per [48]. At passage 2, mixed glial cultures were used as a positive control for GFAP, PU.1, and PDGFRβ immunostaining to compare isolated endothelia in Fig. 1 (Additional file 1: Table S2). Due to the proliferation of pericytes, but not other cell types in mixed glial cultures, serial passaging of mixed glial cultures yields pericyte monocultures, as we have established previously [36]. Pericyte monocultures were used as a negative control for endothelial marker immunostaining in Fig. 2. In experiments where the inflammatory response of endothelial cells and pericytes was compared, only pericyte monocultures were used (Figs. 3, 4, and 5). These were treated as previously described and pericytes used for comparison [51, 52]. Briefly, pericytes were grown in complete DMEM in T75 flasks until confluent. Pericytes were subcultured by trypsinisation (0.25% trypsin-EDTA; Gibco) and seeded at a density of 15,000 cells/cm^2^ for most experiments and 30,000 cells/cm^2^ for ECIS experiments. See Additional file 2: Table S2 for details of epilepsy-derived pericyte monocultures used.

### Isolation and maintenance of brain endothelial cultures

The strainers used to obtain the aforementioned single cell suspension were placed in a sterile petri dish and strainers inverted so that debris could be washed off strainers in complete DMEM. The debris was collected and spun at 170×*g* for 5 min. Media was aspirated, and the pellet was gently resuspended for in ScienCell Endothelial Cell Medium (ECM; ScienCell, CA, USA) plating into a Matrigel® (thin coating 2.5 μL/cm^2^; Corning, NY, USA)-coated flask as per the manufacturer’s instructions (See Table 1 for approximate plating guidelines). Vessels and debris were left to adhere overnight, before being washed gently to detach loosely adhered debris. The media was changed to puromycin (0.5 μg/mL; Sigma-Aldrich, MO, USA)-containing ECM. Elongated, cobblestone-shaped endothelia emerged and could be distinguished from other cell types initially present in cultures, including pericytes (large, stellate), microglia (rhomboidal, phase bright), and astrocytes (complex, thin processes). Human brain endothelial cell cultures were maintained in puromycin-containing ECM for 1–2 weeks in order to eliminate other cell types. Brain endothelia are resistant to puromycin-induced toxicity due to their ability to pump it out, and puromycin therefore selects for endothelial cells [53]. Following this, cells were grown in ECM without puromycin for the remainder of their time in culture. Subculture was achieved by detaching endothelia with StemPro® Accutase® (5 min, 37 °C; Gibco), and plating into new Matrigel®-coated plates at a density of 30,000 cells/cm^2^, except for barrier studies where they were seeded at 60,000 cells/cm^2^ onto collagen I-coated plates (1 μg/cm^2^; Gibco). In general, barrier studies were performed in passages 2–3, while secretion analysis was performed at passages 4–5. Endothelia can be maintained over multiple passages (Range: 3–8 passages) and are amenable to cryostorage in 90% FBS:10% DMSO for extended periods. In order to successfully passage endothelia multiple times, flasks should not be split at a ratio of less than 1:2.

**Table 1:** Approximate guidelines for culture area for a given mass of tissue

Tissue weight	Culture flask
Less than 0.5 g	12-well plate
0.5–2 g	9 cm^2^ Petri dish
2–10 g	T25
More than 10 g	T75

### Matrigel® coating of plates and flasks

Matrigel® (Corning) was allowed to thaw on ice, before being diluted 1:80 in DMEM:F12 without any additives (Gibco) and kept on ice. Ice cold diluted Matrigel® was added to plates at 2.5 μL Matrigel® per square centimetre of plastic and left at room temperature for at least 1 h. Plates and flasks were then washed three times for at least 30 min in additive-free DMEM:F12, before this was aspirated and surfaces allowed to air-dry overnight. Plates and flasks were then sealed and kept at 4 °C for up to 3 months.

### Magnetic-activated cell sorting

For initial attempts to isolate endothelia, magnetic-activated cell sorting (MACS) based on CD31 expression was used (Additional file 1: Figure S1). Briefly, endothelial cells were cultured as above; however, puromycin was omitted. At the first passage, Accutase (Gibco, CA, USA) was used to dissociate cultures, before being washed in MACS buffer (1% bovine serum albumin, 1 mM EDTA in PBS) and Fc receptor blocking agent added, followed by addition of magnetic bead-conjugated mouse IgG1 anti-human CD31 antibody (Miltenyl Biotech, Cologne, Germany) for 15 min at 4 °C. The cell suspension was washed in MACS buffer and passed through a MACS separation column, as per the manufacturer’s instructions to elute the negative fraction, followed by elution of the CD31-positive endothelial fraction in MACS buffer. The positive and negative fractions were spun at 170×*g* for 5 min and plated at a density of 30,000 cells/cm^2^ into 96-well plates.

### Cytokine treatments

Inflammatory cytokines interleukin-1β (IL-1β), tumour necrosis factor-α (TNFα), lipopolysaccaride (LPS), interferon-γ (IFN-γ), transforming growth factor-β1 (TGF-β_1_), interleukin 6 (IL-6), and interleukin 4 (IL-4) or vehicle (a mixture of the vehicles for IL-1β, TNFα, LPS, IFN-γ, IL-6, and IL-4 (0.1% bovine serum albumin (BSA) in phosphate buffered saline (PBS)) and 1 mM citric acid, pH 3 with 0.1% BSA, for TGF-β_1_) were added as a 100-fold dilution of a 100× stock to both pericytes and endothelia. See Table 2 for details.

**Table 2:** Inflammatory stimuli panel used, concentrations, treatment times, and suppliers

Inflammatory stimulus	Concentration (ng/mL)	Incubation	Supplier	Catalogue
IL-1β	10	1 h, 24 h	PeproTech, NJ, USA	200-01B
TNFα	10	1 h, 24 h	PeproTech, NJ, USA	300-01A
LPS (*E. coli*, 026:B6)	10	1 h, 24 h	Sigma, MO, USA	L4391
IFN-γ	10	1 h, 24 h	R&D Systems, MN, USA	285-IF
TGF-β_1_	10	1 h, 24 h	PeproTech, NJ, USA	100-21
IL-6	10	1 h, 24 h	PeproTech, NJ, USA	200-06
IL-4	10	1 h, 24 h	PeproTech, NJ, USA	200-04

### Immunocytochemistry (ICC)

Cells in 96-well plates were fixed in 4% paraformaldehyde for 15 min and washed with PBS with Triton™ X-100 (0.1%; Sigma, MO, USA) (PBS-T). Primary antibodies, diluted appropriately in immunobuffer (PBS with 0.2% Triton™ X-100, 1% goat serum (Gibco, CA, USA) and 0.04% thimerosal (Sigma, MO, USA)), were added overnight at 4 °C (See Additional file 2: Table S3 for details). The following day, secondary antibodies were added at room temperature for 3 h. Hoechst nuclear counterstain (1 μM; Hoechst 33258, Sigma, MO, USA) in Tris-NaCl-EDTA (TNE) buffer was added for 10 min at room temperature. For stains that were sensitive to detergent (zona occludens-1; ZO-1), Triton™ X-100 was excluded until after the primary antibody incubation. Wells were then washed again and counterstained with Hoechst-33258 nuclear stain. For experiments directly comparing endothelial and pericyte inflammatory response, both endothelia and pericytes were seeded and imaged on the same plate. Images were acquired on an ImageXpress Micro XLS™ automated microscope (Molecular Devices).

### 5-Ethynyl-2′-deoxyuridine (EdU) proliferation assay

Proliferation was measured by 5-ethynyl-2′-deoxyuridine (10 μM; Click-iT® EdU AlexaFluor® 647 Imaging Kit; Molecular Probes, CA, USA) incorporation for 48 h at 37 °C, 5% CO_2_. Fixed cells that had been immunostained, as described previously, were then washed in 3% BSA in PBS-T twice. EdU staining was visualised using the Click-iT® AlexaFluor™ 647 kit as per the manufacturer’s instructions. Nuclei positive for EdU were scored using MetaXpress™ version 5.3.04 (Molecular Devices) analysis software.

### Image analysis

Quantification of cell populations was performed by immunocytochemistry with well-characterised lineage markers. Nuclei positive for PU.1 were designated as microglial using MetaXpress™ software, while in mixed glial cultures, CD31-positive cells lacking PU.1-positive nuclei were designated as endothelia. Remaining cell populations could not be accurately scored by automated analysis and were therefore counted manually. Glial fibrillary acidic protein (GFAP)-positive cells were determined to be astrocytes. The remaining cells were found to be platelet derived growth factor receptor-β (PDGFRβ)-positive and designated as pericytes. In endothelial cultures, the remainder of GFAP-negative, PU.1-negative, and PDGFRβ-negative cells were designated endothelia. Quantification of intercellular cell adhesion molecule-1 (ICAM1) and MCP-1 intensity was performed by setting a low threshold and measuring the integrated intensity of the thresholded pixels, relative to the cell number. Quantification of signal transducer and activator of transcription-1 (STAT1) and similar to mothers against dodecapentaplegic-2/3 (SMAD2/3) was performed by setting a high threshold and scoring the percentage of Hoechst-positive nuclei above the threshold value. Quantification of nuclear factor kappa-light-chain-enhancer of activated B cells p65 (NF-κB) translocation was performed using the translocation assay built in to MetaXpress™ software.

### Quantitative real-time polymerase chain reaction (qPCR)

Total RNA was extracted from endothelia or pericytes grown in a 12-well plate using the Ambion RNA Micro-scale extraction kit (Ambion, CA, USA) following the manufacturer’s instructions. For complementary DNA (cDNA) synthesis, 500 ng total cDNA was made per sample. RNA was added to each reaction mix accordingly and diluted to give the same volume, before deoxyribonuclease I (DNase I) from the RQ1 RNase-free DNase kit (Promega, WI. USA) with 1 μg DNase added per 1 μg RNA. The Superscript III First-Strand Synthesis kit (Life Technologies, CA, USA) was used for cDNA synthesis. Quantitative real-time PCR was performed using Platinum SYBR Green qPCR SuperMix-UDG with Rox (Life Technologies, CA, USA) on a 7900HT Fast Real-Time PCR system (Applied Biosystems, Life Technologies, CA, USA) (see Additional file 1: Table S4 for primer details). Relative gene expression was graphed as fold enrichment using the 2^−ΔΔCt^ method compared to RNA obtained from a single culture of pure pericytes [29].

### Electrical cell-substrate impedance sensing (ECIS) for barrier analysis

Electrical cell-substrate impedance sensing (ECIS) is a technique used to measure the resistance to flow of current across a monolayer of cells at multiple frequencies in real time. To determine the barrier properties of isolated human brain endothelial cells or pericytes, experiments were performed on collagen I-coated (1 μg/cm^2^; Gibco, CA, USA) ECIS 96W20idf plates (0.33 cm^2^ well; Applied BioPhysics, NY, USA) for the ECIS Z ϴ instrument (Applied BioPhysics, NY, USA). Endothelia were then seeded at 60,000 cells/cm^2^ and generated raw impedance values of approximately 2000–2500 Ω when measured at 4000 Hz. Cells were cultured until transendothelial electrical resistance (TEER) had plateaued (between 96 and 120 h post plating) before treatments were performed. Barrier resistance values were obtained using ECIS software (Version 1.2.123.0; Applied Biophysics, NY, USA), using the modelling method described by Giaever and Keese [54], and applied to endothelial barrier measurements elsewhere [55]. Briefly, the changes in impedance relative to a cell-free, media-containing well were measured and used to determine the components of impedance defined by cell shape/granularity (capacitance, measured in nF), cell-substrate contacts (alpha, measured in Ω^-1/2^/cm), and cell-cell contacts (R_b_, given as TEER in results, measured in Ω cm^2^). It should be noted that ECIS does not detect comparable levels of resistance to those generated by an EVOM voltohmmeter, which is commonly used to measure TEER, where the units for are in Ω cm^−2^, rather than Ω cm^2^; however, the measurements represent similar properties of the monolayer.

### Cytometric bead array (CBA)

Conditioned media samples from endothelial and pericyte cases were spun at 160×*g* for 5 min and the supernatant collected and stored at − 20 °C. The concentration of cytokines was measured using a cytometric bead array (CBA; BD Biosciences, CA, USA) as per manufacturer’s instructions (see Table 3 for CBA kit details). CBA samples were run on an Accuri C6 flow cytometer (BD Biosciences, CA, USA). Data were analysed using FCAP-array software (version 3.1; BD Biosciences, CA, USA) to convert fluorescent intensity values to concentrations using an 11-point standard curve (0–10,000 pg/mL) and normalised to cell number as described previously [52, 56].

**Table 3:** CBA kits and specifications used in these studies

Antibody	Cat no.	Bead position
sCD54/ICAM-1 (intercellular adhesion molecule-1)	560269	A4
sCD106/VCAM-1 (vascular cell adhesion molecule-1)	560427	D6
Fractalkine (CX3CL1)	560265	C6
G-CSF (granulocyte-colony stimulating factor)	558326	C8
GM-CSF (granulocyte macrophage-colony stimulating factor)	558335	C9
IL-6 (interleukin-6)	558276	A7
IL-8 (interleukin-8)	558277	A9
IP-10 (interferon gamma-induced protein-10)	558280	B5
MCP-1 (monocyte chemoattractant protein-1)	558287	D8
RANTES (regulated on activation, normal T cell expressed and secreted)	558324	D4

### Secretome profiler analysis

Conditioned media samples from two independent endothelial and pericyte cases treated with either vehicle or IL-1β (10 ng/mL, 24 h) were spun at 160×*g* for 5 min, the supernatant was collected and stored at − 20 °C. Conditioned media was assayed using the Proteome Profiler™ Human XL Cytokine Array Kit as per the manufacturer’s instructions (R&D Systems, MN, USA). Briefly, membranes spotted with antibodies were incubated with conditioned media overnight at 4 °C. The following day, detection antibody cocktail was added for 1 h at room temperature, before visualisation using chemiluminescence. Images were acquired using the Li-COR Odyssey FC® imaging system, and spot intensity was quantified using ImageJ and normalised to the reference spots.

### Statistical analysis

All experiments were performed in at least three independent cases. In general, data are expressed as mean ± standard error of mean (SEM) from at least three independent experiments. Data visualisation and statistical hypothesis testing was performed using GraphPad Prism® Version 7.00. Two-way analysis of variance (ANOVA) was used when comparing cell type response across a number of stimuli with Tukey’s post hoc adjustment for multiple comparisons, while one-way ANOVA with Dunnett’s multiple comparison adjustment was used when comparing changes in TEER in response to stimuli. For heatmap generation, including unbiased hierarchical clustering, the heatmap.2 function was utilised in ‘R’ software.

## Results

### Determination of endothelial purity

After having obtained large debris from tissue dissociation (Fig. 1a–k), blood vessels containing viable endothelia were isolated due to their ability to stick to the Matrigel®-coated plastic. Endothelia were seen to emerge from vessels within 24 h (Fig. 1i). Visual inspection of cultures indicated that although microglia (Fig. 1i, asterisks) and pericytes (Fig. 1i, stars—only observed attached to vessels) were present early in endothelial cultures, they were eliminated within a week due to the toxicity of puromycin. Earlier attempts at isolating endothelia by cell sorting, but without puromycin, had a high degree of pericyte contamination (Additional file 1: Figure S1), and so it is recommended that puromycin treatment be continued for an additional week, and only stopped 1 or 2 days prior to the first passage. Endothelial cultures have a different morphology to mixed glial cultures, with a cobblestone monolayer of spindle-shaped cells (Fig. 1i; endothelial colonies outlined in red). On the other hand, mixed glial cultures contained astrocytes (Fig. 1i, arrows), microglia (asterisks), and pericytes (stellate, phase dark cells, prominent 1 week post plating). The purity of cultures was assessed by staining for markers of endothelia (CD31), microglia (PU.1), astrocytes (GFAP), and pericytes (PDGFRβ). The purity of endothelial cultures was determined to be greater than 99% (Fig. 1m, n). In only one culture, a small population of contaminating pericytes was observed, while no microglia or astrocytes were present. In mixed glial cultures, a population of CD31-positive cells were seen, albeit with weak CD31 staining; however, these were predominantly PU.1-positive and thus categorised as microglia. CD31-positive and PU.1-negative cells were also seen in mixed cultures and denoted as endothelia, though this comprised less than 1% of the population (Fig. 1m, n).

### Isolated endothelia are proliferative and re-establish blood-brain barrier phenotype

Brain endothelial cells are notable for their high expression of tight and adherens junction proteins, and transporters, and these are of considerable interest in the study of the BBB [4]. Thus, we assessed the presence of the tight junction proteins ZO-1 and claudin-5, along with the adherens junction protein CD31, and endothelial nuclear marker ETS-related gene (ERG). All isolated endothelial cultures expressed these proteins, and staining was not present in negative control pericyte cultures (Fig. 2a). Staining patterns also indicated that ZO-1 was expressed at the junctions of cells, alongside claudin-5 staining (Fig. 2b). Likewise, CD31 expression was higher at the borders of cells, but more widely distributed across endothelia (Fig. 2c). In addition, qPCR studies revealed that genes encoding junctional proteins claudin-5, CD31, occludin, vascular endothelial cadherin (VE-cadherin), transporters glucose transporter-1 (GLUT-1), transferrin receptor (TfR), and P-glycoprotein (P-gp) and endothelial-specific von Willebrand Factor (vWF) were enriched in endothelial cultures, relative to pure pericyte cultures (Fig. 2d). Endothelial expression of the gene encoding the pericyte-specific protein for PDGFRβ was also 1000-fold lower than pericyte cultures, indicating that cultures have minimal pericyte contamination. We also observed that endothelia are proliferative in vitro, as shown by EdU*-*incorporation (Fig. 2e, f), and this can be increased with VEGF-A treatment (Additional file 1: Figure S2).

### Inflammatory responses through the NF-κB, STAT1, and SMAD2/3 pathways are conserved between endothelia and pericytes

We assessed the response of endothelia and pericytes to a panel of immune mediators (IL-1β, TNFα, LPS, IFN-γ, TGF-β_1_, IL-6, and IL-4) by examining the activation of the transcription factors NF-κB, STAT1, and SMAD2/3 after 1 h of treatment and the immunostaining of ICAM-1 and MCP-1 after 24 h of treatment. Treatment with IL-1β, TNFα, and LPS caused translocation of NF-κB to the nucleus, with no significant differences observed between endothelia and pericytes (Fig. 3a, b). IFN-γ caused the translocation of STAT1 to the nucleus, with no significant differences observed between the cell types (Fig. 3c, d). SMAD2/3 translocation occurred in response to TGF-β_1_ stimulation in pericytes and endothelial cells, although endothelial cells displayed higher basal nuclear SMAD2/3 than pericytes (Fig. 3e, f). No changes in transcription factor localisation were observed following stimulation with IL-4 or IL-6. Representative images from all conditions are given in Additional file 1: Figure S3.

### Endothelia and pericytes have differential secretion profiles in response to immune stimuli

Using cytometric bead array (CBA), we tested the secretions of soluble ICAM-1 (Fig. 4a, k), soluble VCAM-1 (Fig. 4b, i), G-CSF (Fig. 4c, m), GM-CSF (Fig. 4d, n), CX3CL1 (Fig. 4e, o), IL-6 (Fig. 4f, p), IL-8 (Fig. 4g, q), MCP-1 (Fig. 4h, r), IP-10 (Fig. 4i, s), and RANTES (Fig. 4j, t) from endothelia and pericytes in response to the inflammatory panel. Both endothelia and pericytes have a similar response profile to the immune stimulus panel, with slightly higher levels of ICAM-1 secreted by endothelia in response to IL-1β and TNFα. We also detected soluble ICAM-1 in vehicle-treated endothelia, but not pericytes, consistent with ICC data (Additional file 1: Figure S4). On the other hand, CX3CL1 and VCAM-1 were basally secreted at higher levels in pericytes compared to endothelia, but regulated similarly in both endothelia and pericytes in response to the inflammatory stimuli (Fig. 4b, e, i, o). Pericyte secretion of CX3CL1 was also suppressed in response to TGF-β_1_ and IL-4 (Fig. 4e, o). Both G-CSF and GM-CSF had similar secretion patterns, being strongly induced in endothelia, but not pericytes (Fig. 4c, d, m, n). Interestingly, in endothelia, IL-1β induced 5–10-fold more G-CSF and GM-CSF than the next strongest inducer, LPS. Both pericytes and endothelial cells secreted IL-6 in response to IL-1β stimulation, with endothelia demonstrating a stronger induction than pericytes (Fig. 4f, p). While the clearest induction of IL-6 occurred in response to IL-1β, it was also induced in both endothelia and pericytes in response to TNFα and LPS. IL-8 secretion was strongly induced by IL-1β, TNFα, and LPS in both endothelia and pericytes, though endothelial secretion of IL-8 in response to TNFα was weaker than pericytes (Fig. 4g, q). Similar levels of RANTES secretion were observed in response to LPS in both endothelia and pericytes, although endothelia secreted greater levels in response to IL-1β and TNFα (Fig. 4j, t). Interestingly, IL-4 induced a greater degree of VCAM-1 and MCP-1 secretion in pericytes than endothelia (Fig. 4h, r). Likewise, pericytes secreted higher concentrations of MCP-1 and ICAM-1 in response to IFN-γ than endothelia (Fig. 4a, k, h, r); however, endothelia secreted greater levels of IL-6 and IP-10 than pericytes (Fig. 4p, s). For a full summary of responses, see Table 4.

**Table 4:** Summary of endothelial and pericyte secretions in response to inflammatory stimuli used in this study

	ICAM-1		VCAM-1		CX3CL1		GM-CSF		G-CSF		IL-6		IL-8		IP-10		MCP-1		RANTES	
	Endo	Peri	Endo	Peri	Endo	Peri	Endo	Peri	Endo	Peri	Endo	Peri	Endo	Peri	Endo	Peri	Endo	Peri	Endo	Peri
Vehicle	+	–	–	+	–	++	–	–	–	–	–	–	+	+	–	–	+	+	–	–
IL-1β	++++	+++	++	++	++++	+++	++++	+	++++	–	++++	+++	++++	++++	+	–	+++	+++	++	+
TNFα	++++	+++	+++	++++	++++	+++	+	–	+	–	+	++	++	++++	+	–	+++	++++	+	+
LPS	+++	+++	++	+++	+++	+++	++	–	++	–	+++	++	+++	+++	+++	+	+++	+++	++	++
IFN-γ	+	+	–	+	+	++	–	–	–	–	–	+	+	+	++++	++	+	+++	–	–
TGF-β_1_	+	–	–	+	–	–	–	–	–	–	–	+	+	+	–	–	+	+	–	–
IL-6	+	–	–	+	–	++	–	–	–	–	+++	+++	+	+	–	–	+	+	–	–
IL-4	+	–	+	++	–	+	–	–	–	–	–	+	+	+	–	–	+	++	–	–

### Endothelia and pericytes have overlapping but distinct secretions in response to IL-1β stimulation

Corroborating CBA and immunocytochemistry data, secretions in endothelial and pericyte conditioned media were found to be strongly altered by IL-1β treatment. Both cell types demonstrated induction of a wide range of inflammatory mediators, as measured by secretome profiler array (Fig. 5a, b, d). The majority of the secretions assessed here are similar between the two cell types, with 75 secretions shared (Fig. 5d). There were 15 unique endothelial secretions including soluble CD31, the marker for endothelial cells used above (Fig. 5e). We also identified the secretion of two unique pericyte secretions, insulin-like growth factor binding protein-3 (IGFBP-3) and CD40L, the expression of which have been shown in pericytes and smooth muscle cells previously [56, 57] (Fig. 5e). Interestingly, the IGF-1 binding proteins IGFBP-2 and IGFBP-3 are highly expressed by pericytes, but not endothelia. This experiment was performed twice across two different cases; however, there was a high degree of consistency between the two sets of results (*R*^2^ = 0.82; Fig. 5c). An additional file provides intensity measures for all cytokines using secretome profiler arrays (Additional file 2).

### Endothelial barrier integrity is strongly reduced by inflammatory stimuli

**Fig. 6:**
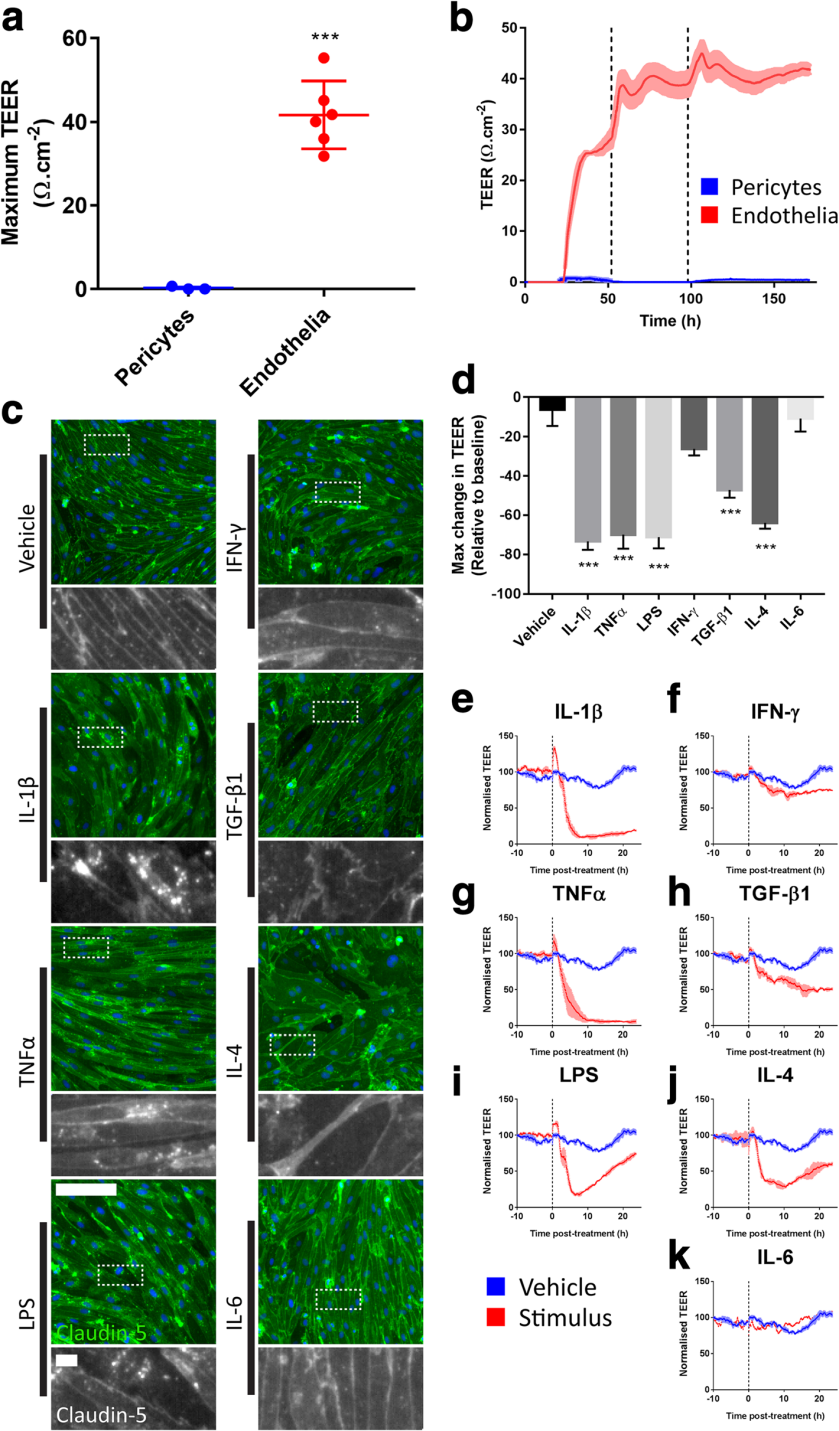
Isolated endothelia generate high transendothelial electrical resistance (TEER), which is reduced by application of cytokines. Endothelia or pericytes were grown on an ECIS array until TEER had plateaued (96 – 120 h), then treated with either vehicle, IL-1β, TNFα, LPS, IFN-γ, TGF-β_1_, IL-4, or IL-6 (10 ng/mL) for a further 24 h and tight junctions stained by immunocytochemistry. **a**) Endothelial cultures generate TEER in excess of 35 Ω.cm2 (Mean ± SEM from 6 independent cases). **b**) Representative ECIS trace from primary endothelia and pericytes. Dashed lines denote when media was changed. Mean ± SEM from duplicate wells. **c**) Representative images of claudin-5 immunostaining in endothelia treated with immunogens. Scale bar = 100 μm, inset = 5 μm. **d**) Maximal change in TEER relative to 10 h pre-treatment baseline in endothelia treated with inflammatory stimuli. Mean ± SEM, *n* = 3 - 4 independent experiments across 2 – 3 cases. **e** - **k**) Representative traces from endothelial cultures treated with inflammatory stimuli (red) overlayed on vehicle-treated endothelia (blue) (mean ± SEM from duplicate wells). Dashed lines denote the point the treatment was added

Inflammation is a critical regulator of the BBB in vivo, so continuous measurement of TEER in primary brain endothelia and pericytes using ECIS was performed to assess functional barrier integrity, alongside immunocytochemistry to test if this was accompanied by a reorganisation of tight junctions. Primary endothelial cells generated consistently high TEER, with maximal TEER between 35 and 55 Ω cm^−2^ with raw impedance of approximately 2500 Ω (Additional file 1: Figure S5), unlike pericytes (range 0.1–1 Ω cm^−2^, raw impedance approximately 1000 Ω) where barrier resistance was not a large component of the resistance to current (Fig. 6a, b). Measurements indicated that TEER plateaued after day 4 (Fig. 6b). The TEER of primary brain endothelial cultures was rapidly reduced by between 60 and 75% following application of IL-1β, TNFα, LPS, and IL-4 (Fig. 6d, e, g, i, j), with IL-4 and LPS-treated cells showing a rebound effect. We found that treatment of endothelia, but not pericytes, with IL-1β and TNFα resulted in toxicity (Additional file 1: Figure S4). Treatment with TGF-β_1_ also reduced TEER by approximately 50% (Fig. 6d, h). Neither IFN-γ nor IL-6 had any significant effect on TEER (Fig. 6d, f, k). These data were reinforced by claudin-5 staining which revealed a reorganisation of tight junctions to granules in cells treated with IL-1β, TNFα, and LPS (Fig. 6c). In cells treated with LPS and TGF-β_1_, discontinuous unsealed claudin-5 staining was present, while in IL-4-treated endothelia claudin-5-positive tight junctions appeared to have more diffuse staining (Fig. 6c). In TNFα and IL-4-treated endothelia, there was also a notable change in cell morphology to more spindle-shaped and more rounded, respectively (Fig. 6c).

## Discussion

Here, primary brain endothelial and pericyte cultures were used to study inflammatory processes at the BBB. The BBB is a critical target of central nervous system inflammation, and we show overlapping, but distinct, responses of two key cell types in the BBB, endothelia, and pericytes. Importantly, the protocol used to derive endothelial cells was adapted to work in concert with other brain cell culture protocols from our laboratory [48–50], without requiring additional tissue. While tissue was derived from diseases that have a strong neuroinflammatory and neurovascular component [18, 19], we observed that a consistent phenotype and response profile was maintained, as has been reported in our pericyte cultures previously [36, 58, 59]. Reinforcing this is the observation that markers of inflammatory activation in both cell types were low or absent under control conditions. Likewise, in endothelial cells, despite the derivation of cells from numerous tissue sources, consistent responses and a robust BBB phenotype were observed, with no obvious patterns associated with diseases.

**Fig. 7:**
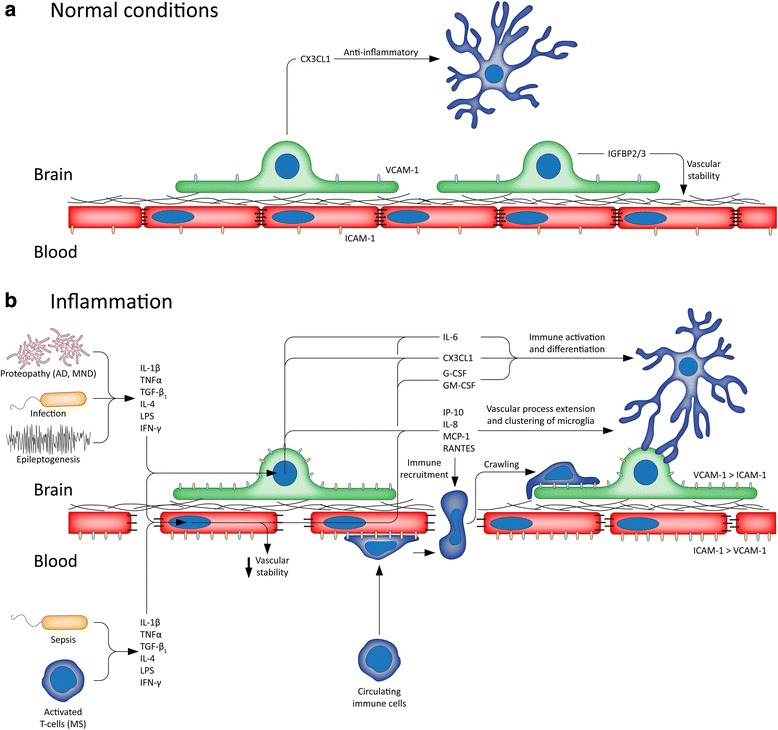
Contributions of vascular inflammation to neuroinflammatory processes. **a** Under normal conditions pericyte secretion of CX3CL1 and IGFBP2 and IGFBP3 may be important to the regulation of inflammation and vascular stability. Pericytes basally express low levels of VCAM-1, while endothelia express ICAM-1. **b** The neurovasculature can be affected by inflammation arising in the brain (e.g. epilepsy, infection, AD) or the periphery (e.g. MS, sepsis). Inflammation disrupts BBB stability by acting on endothelia. Cytokines also activate both pericytes and endothelia to secrete chemoattractants, and upregulate ICAM-1 and VCAM-1 expression. These encourage the recruitment and infiltration of peripheral immune cells through the weakened BBB, where IL-6, CX3CL1, G-CSF, and GM-CSF determine their differentiation and activation. Chemoattractants also encourage the recruitment of microglia and their processes to the vasculature, leading to vascular microglial clustering

Examining BBB properties of endothelia in vitro revealed that many of the inflammatory stimuli examined had detrimental effects on TEER. As expected, endothelia, but not pericytes, formed strong enough cell-cell contacts to generate robust TEER, with pericytes generating approximately 0.2 Ω cm^−2^ of TEER, compared to in excess of 40 Ω cm^−2^ in endothelia. Due to the type of ECIS arrays used, all treatments were added to the in vitro equivalent of the luminal side of endothelia, mimicking blood-borne inflammatory signals. Signalling through the NF-κB pathway by IL-1β, TNFα, and LPS caused a rapid reduction in TEER; however, there is a recovery following LPS stimulation. At the concentrations of IL-1β and TNF-α used, toxicity to endothelia, but not pericytes, was observed by a reduction in cell number. This toxicity likely accounts for the prolonged loss of barrier integrity as a result of IL-1β and TNFα treatment. However, the rapid onset of the IL-1β and TNFα effects cannot be explained solely by toxicity. Stimulation with IL-4 also led to loss of barrier integrity, indicating that, despite having an anti-inflammatory function, its presence is also detrimental to the BBB, something that has not been observed previously. This is a clear demonstration that anti-inflammatory actions of cytokines are not necessarily synonymous to positive effects on the BBB. Stimulation of endothelia with TGF-β_1_ also reduced barrier integrity; however, this was weaker than the activators of NF-κB, and IL-4. Overall, these results indicate that inflammation with its genesis in the periphery or the brain can drive BBB opening through a number of different cytokines. Indeed, these data provide insight into the connection between inflammation and BBB breakdown in diseases such as epilepsy [18, 19], multiple sclerosis [24, 25], and Alzheimer’s disease [60] (Fig. 7).

Unsurprisingly, the responses of both endothelia and pericytes to the immune stimulus panel were similar, indicating a concerted response to inflammatory stimuli. While other studies have examined endothelial [61–63] and pericyte [36, 37] responses to a limited panel of stimuli, this is the first direct comparison of primary human brain endothelial and pericyte inflammatory responses to such a wide range of stimuli, and in such detail. Despite the large overlap in responses, we found that the effects of IL-4 and TGF-β_1_ on pericyte secretions were stronger than on endothelia. In pericytes, both IL-4 and TGF-β_1_ upregulated IL-6 and downregulated CX3CL1, but had opposing effects on MCP-1 and VCAM-1. This corroborates previous findings from our group, indicating that TGF-β_1_ activates inflammatory processes in pericytes [56]. The suppression of CX3CL1 and induction of MCP-1, IL-6, and VCAM-1 in response to IL-4 suggest that its actions may enhance pericyte inflammatory signals, in contrast with its well-known anti-inflammatory effects on immune cells [64]. We did not observe the activation of NF-κB, STAT1, or SMAD2/3 pathways by IL-4, though it is reported to predominantly act through STAT5 [65], which is likely to be responsible for the changes to secretions and barrier function of cells that was observed. Interestingly, IL-6 did not appear to modulate any pathways or secretions in either cell type, nor did it alter barrier function in endothelial cells. Secretion data also indicated that, in general, TNFα was the strongest inducer of inflammatory responses in pericytes, consistent with previous results [61], whereas IL-1β produced the strongest response in endothelia. We found that both endothelia and pericytes could be induced to secrete CX3CL1, although pericytes secreted higher levels of it at baseline. This is potentially important, as the anti-inflammatory action of CX3CL1 is restricted to microglia, which selectively express the receptor CX3CR1 [66]. These data confirm previous reports from our group [56], that pericytes are a source of CX3CL1 in the brain, and here, we show that endothelia are another source of CX3CL1 at the vasculature.

We show here that responses to IL-1β, TNFα, and LPS through NF-κB were conserved between cell types, as well as response to IFN-γ through STAT1, and to TGF-β1 through the SMAD2/3 pathway. Despite this, constitutively elevated nuclear SMAD2/3 was observed in endothelia, unlike pericytes, which aligns with the finding that SMAD2/3 is essential to endothelial stability in vivo [67]. Using ECIS, TGF-β_1_-mediated disruption of the BBB was observed, but despite this, we did not observe significant alterations in endothelial secretions. This may be due to technical considerations that were not investigated here, such as the time the secretions were measured, or may represent differences in TGF-β1 signalling between the cell types. We hypothesise that differential responses may be due to the endothelial-specific expression of the TGF-β_1_ receptor, activin like kinase-1 (ALK1) [68]. ALK1 signals through the SMAD1/5/8 pathway [69] and may alter the outcome of TGF-β_1_ treatment.

We found that G-CSF and GM-CSF were highly, and almost exclusively, secreted by endothelia. Both G-CSF and GM-CSF are important cytokines regulating the differentiation, activation, and survival of immune cells [70, 71]. Generally, GM-CSF has been found to promote inflammation [70], while G-CSF has been found to suppress it [72–74]. In situ hybridisation of EAE and IL-1β-treated mice has revealed that GM-CSF, IL-6, and G-CSF are expressed at the vasculature, and were found to be upregulated in the conditioned media of stimulated endothelial cells [25]. Interestingly, GM-CSF has previously been shown to enhance monocyte transmigration of endothelia in vitro and increase expression of MHC-II, CD40, TNFα, and reactive oxygen species production [75]. In line with these observations is the recent finding that IL-1β selectively drives G-CSF and GM-CSF expression in endothelia, and that it is required for the transmigration of monocytes in EAE by upregulating CCR2 [24]. Furthermore, IL-1β causes monocytes to differentiate into antigen presenting cells in the perivascular space, priming infiltrating T-cells [24]. In addition, IL-1β expression by bone marrow-resident myeloid cells was required for rodents to develop EAE [24]. This work complements these findings that G-CSF and GM-CSF expression is restricted to endothelial cells and highlights the importance of these factors in regulating the transmigration of immune cells. The roles of G-CSF, and many other vascular secretions here, are less well known, and warrant further study.

Results from CBA analysis revealed that IL-1β was the strongest inflammatory stimulus overall, inducing the expression of every secretion measured by cytometric bead array. The difference in IL-1β response in pericytes and endothelia observed using the secretome profiler reinforced CBA data. Secretome profiling also detected that VCAM-1 is constitutively secreted by pericytes, while GM-CSF and G-CSF are strongly secreted by endothelia. Despite this, the majority of secreted factors from endothelia and pericytes were shared, differing only in the level of secretion. Amongst the most highly expressed secretions in both cell types were Serpin E1, GRO-α, MIP-3α, CXCL5, IL-8, IL-6, and MCP-1. Both cell types strongly secreted a diverse range of chemoattractants, which corresponds well with the role of the cerebrovasculature in the recruitment of immune cells upon inflammatory insult. These chemoattractants may also be relevant to vascular clustering of microglia [11, 18] or microglial process extension following insult [76]. We identified several unique or highly enriched endothelial secretions that were not measured by CBA, including angiopoietin-2, CD31, RAGE, and PDGF-AB/BB. In contrast, pericytes, but not endothelia, secreted high levels of IGFBP-2, and IGFBP-3, something that has not been shown previously. This is particularly interesting, since IGFBPs, especially IGFBP3, have been found to be involved in endothelial viability [77, 78], angiogenesis [79], inflammation, and pericyte coverage [80], as well as being regulated by amyloid-β in pericytes [81].

While similarities between endothelial and pericyte inflammatory responses are expected, the differences in responses potentially highlight divergent roles for the two cell types in the regulation of the neurovascular response to inflammation. This study highlights more differences between endothelia and pericytes than have been shown previously [61], as we used a wider range of inflammatory stimuli. We also highlight a number of unique secretions, produced in unstimulated endothelia and pericytes, which may shed light on how pericytes and endothelia regulate signalling at the BBB in different ways. We detected unique secretions in each cell type, representing a unique response to inflammatory stimuli in primary human brain endothelia and pericytes for the first time. A number of these secretions are known to be critical regulators of the immune response in the brain, especially G-CSF, GM-CSF, and CX3CL1. Indeed, it will be important to examine how inflammation shapes the interactions of endothelia and pericytes, and how the secretions identified here may shape these. These findings highlight that the cells of the BBB are active players in neuroinflammatory processes. Taken together, these data represent cell type-specific targets in pericytes and endothelia that may be involved in BBB homeostasis, as well as providing further evidence that the neurovasculature is an active player in neuroinflammation (Fig. 7).

## Conclusions

Pericytes and endothelia show unique response profiles in response to a range of inflammatory stimuli and reinforce the active role that the neurovasculature plays in shaping neuroinflammation.

## Additional files

Additional file 1: Supplementary material. **Table S1.** Case details for tissue used to culture endothelial cells used in this study. **Table S2.** Case details for pericyte cultures used in this study. **Table S3.** Specifications of antibodies used in this study. **Table S4.** Sequences and targets of primers used in this study. Figure S1 Pericyte contamination develops in endothelial cultures in the absence of puromycin selection following cell sorting. **Figure S2.** Brain endothelial cultures are proliferative and have a proliferative response to VEGF-A. **Figure S3.** Representative images showing the pathways activated by inflammatory stimuli in pericytes and endothelial cells, quantified in Fig. 3. **Figure S4.** Inflammatory response of endothelia and pericytes to the inflammatory stimulus panel analysed by immunocytochemistry **Figure S5.** Brain endothelia generate robust barrier properties in vitro. (DOCX 5909 kb)

Additional file 2: Full secretome profiler dataset from Fig. 5. Pericytes or endothelia from two different cases were treated with either vehicle, or IL-1β (10 ng/mL) for 24 h before media were harvested and secretions analysed as per Fig. 5. Values represent intensity measurements from each secretion spot (two spots per secretion) from both biological replicates, from all treatment groups (Vehicle/Pericyte, IL-1β/Pericyte, Vehicle/Endothelial, IL-1β/Endothelial). Data are given in an Excel spreadsheet. (XLSX 41 kb)
