# Supplementary material for: Unique and shared inflammatory profiles of human brain endothelia and pericytes
Source: J Neuroinflammation. 2018 May 11;15:138. doi: 10.1186/s12974-018-1167-8 (PMC5948925; doi:10.1186/s12974-018-1167-8)
Supplement: Supplementary file 1 — Supplementary material. Table S1. Case details for tissue used to culture endothelial cells used in this study. Table S2. Case details for pericyte cultures used in this study. Table S3. Specifications of antibodies used in this study. Table S4. Sequences and targets of primers used in this study. Figure S1 Pericyte contamination develops in endothelial cultures in the absence of puromycin selection following cell sorting. Figure S2. Brain endothelial cultures are proliferative and have a proliferative response to VEGF-A. Figure S3. Representative images showing the pathways activated by inflammatory stimuli in pericytes and endothelial cells, quantified in Fig. 3. Figure S4. Inflammatory response of endothelia and pericytes to the inflammatory stimulus panel analysed by immunocytochemistry Figure S5. Brain endothelia generate robust barrier properties in vitro. (DOCX 5909 kb) [file 12974_2018_1167_MOESM1_ESM.docx]

**Isolation of primary human brain endothelia reveals a unique inflammatory profile compared to brain pericytes**

Leon CD Smyth^1,2^, Justin Rustenhoven^1,2^, Thomas I-H Park^1,2,3^, Patrick Schweder^2,4^, Deidre Jansson^1,2^, Peter A Heppner^2,4^, Simon J O’Carroll^2,3^, Edward W Mee^2,4^, Richard LM Faull^2,3^, Maurice Curtis^2,3^ Mike Dragunow^1,2^

**Supplementary material**

**Table S1: Case details for tissue used to culture endothelial cells used in this study.**

| Case | **Pathology** | **Region** | **Age** | **Sex** | **POD/PMD** |
| --- | --- | --- | --- | --- | --- |
| E210 | Epilepsy, grade 3 mesial temporal sclerosis | Middle and inferior temporal gyri | 30 | M | 0 |
| E211 | Epilepsy, mesial temporal sclerosis, ungraded | Middle temporal gyrus,  Hippocampus | 51 | F | 0.5 |
| E213 | Epilepsy, patchy gliosis, no cortical dysplasia, no neoplasia, presumed hippocampal sclerosis | Middle temporal gyrus,  Hippocampus | 23 | M | 0.5 |
| SS37 | Cortical dysplasia | Left cortical | 9 | M | 0.5 |
| SS42 | Cortical dysplasia | Right temporal lobe  Right hippocampus | 8 | M | 1 |
| SS48 | Cortical dysplasia | Cortical specimen | 10 | F | 0.5 |
| T131 | Tumour | Cerebellum | 20 | M | 0 |
| T138 | Tumour | Frontal lobe | 30 | M | 0 |
| T147 | Tumour | Peritumoral regions | 47 | F | 0 |
| AZ115 | AD | Middle temporal gyrus, Cerebellum | 79 | F | 11.5 |
| AZ117 | AD | Middle temporal gyrus | 78 | M | 22 |
| AZ121 | AD | Middle temporal gyrus, Cerebellum | 94 | F | 11.5 |

**Table S2: Case details for pericyte cultures used in this study.**

| Case | **Pathology** | **Region** | **Age** | **Sex** | **POD/PMD** |
| --- | --- | --- | --- | --- | --- |
| E203 | Epilepsy, grade 1 mesial temporal sclerosis | Middle temporal gyrus | 46 | F | 0.5 |
| E204 | Epilepsy, hippocampal sclerosis ILAE type 2 | Middle temporal gyrus | 45 | F | 0.5 |
| E206 | Epilepsy, grade 3 mesial temporal sclerosis | Middle temporal gyrus | 29 | F | 0.5 |

**Table S3: Specifications of antibodies used in this study.**

| Antibody (Clone) | **Company** | **Species** | **Catalogue #** | | **ICC** |
| --- | --- | --- | --- | --- | --- |
| Primary antibody | | | | | |
| ZO-1 (1A12) | Invitrogen | Mouse | 339100 | | 1:500 |
| Claudin-5 (polyclonal) | Abcam | Rabbit | ab131259 | | 1:5000 |
| CD31 (JC70A) | Dako | Mouse | M0823 | | 1:500 |
| PU.1 (9G7) | Cell Signalling | Rabbit | 2258 | | 1:500 |
| PDGFRβ (28E1) | Cell Signalling | Rabbit | 3169 | | 1:500 |
| ICAM1 (15.2) | Santa Cruz | Mouse | sc-107 | | 1:500 |
| MCP-1 (polyclonal) | Abcam | Rabbit | ab9669 | | 1:500 |
| αSMA (1A4) | Dako | Mouse | iS611 | | 1:10 |
| GFAP (polyclonal) | Abcam | Chicken | ab4674 | | 1:5000 |
| NF-κB p65 (polyclonal) | Santa Cruz | Rabbit | sc-372 | | 1:500 |
| STAT1 (D1K9Y) | Cell Signalling | Rabbit | 14994 | | 1:500 |
| SMAD2/3 (C-8) | Santa Cruz | Mouse | sc-133098 | | 1:500 |
| Secondary antibody | | | | | |
| Goat anti-rabbit Alexa 488 | Life Technologies | Goat | | A11008 | 1:500 |
| Goat anti-rabbit Alexa 594 | Life Technologies | Goat | | A11012 | 1:500 |
| Goat anti-mouse Alexa 488 | Life Technologies | Goat | | A11001 | 1:500 |
| Goat anti-mouse Alexa 594 | Life Technologies | Goat | | A11005 | 1:500 |
| Goat anti-mouse Alexa 647 | Life Technologies | Goat | | A21235 | 1:500 |
| Goat anti-chicken Alexa 647 | Life Technologies | Goat | | A21449 | 1:500 |

**Table S4: Sequences and targets of primers used in this study.**

| Accession number | **Gene**  **(Protein)** | **Sequence** | | **Amplicon size (bp)** |
| --- | --- | --- | --- | --- |
| NM_002046.4 | *GAPDH*  (GAPDH) | Fw | CATGAGAAGTATGACAACAGCCT | 113 |
|  |  | Rv | AGTCCTTCCACGATACCAAAGT |  |
| NM_002609.3 | *PDGFRB*  (PDGFRβ) | Fw | CGCAAAGAAAGTGGGCGGCT | 101 |
|  |  | Rv | TGCAGGATGGAGCGGATGTGGT |  |
| NM_000442.4 | *PECAM1*  (CD31) | Fw | AAAGCTGTCCCTGATGCCGT | 80 |
|  |  | Rv | TCTGGCCTTGCTGTCTAAGTTC |  |
| NM_000552.4 | *VWF*  (von Willebrand factor) | Fw | TTGACGGGGAGGTGAATGTG | 85 |
|  |  | Rv | AGCAGAATGATGTACCGGCC |  |
| - NM_001130861.1 | *CLDN5*  (Claudin-5) | Fw | CCCCCGAACCTTCAAAGAGG | 92 |
|  |  | Rv | TCTTGGCCCCAGTCCGTTT |  |
| NM_001795.4 | *CDH5*  (VE-Cadherin) | Fw | TCTTCACCCAGACCAAGTACAC | 93 |
|  |  | Rv | GCTCATCTGGGTCCTCAACAA |  |
| NM_001348945.1 | *ABCB1*  (P-glycoprotein) | Fw | GCCAAAATATCAGCAGCCCA | 100 |
|  |  | Rv | TTCCTTCCAATGTGTTCGGC |  |
| NM_002538.3 | *OCLN*  (Occludin) | Fw | CGAGCGGATTGGTTTATCTTGG | 87 |
|  |  | Rv | CCTGGATGACATGGCTGATTG |  |
| NM_006516.2 | *SLC2A1*  (GLUT1) | Fw | TGGCATCAACGCTGTCTTCT | 92 |
|  |  | Rv | CGATACCGGAGCCAATGGTG |  |
| NM_001063.3 | *TFRC*  (Transferrin receptor) | Fw | TGCTCCACCTCATCACTCCT | 94 |
|  |  | Rv | TCTGCGTTCCCATCTTCACC |  |


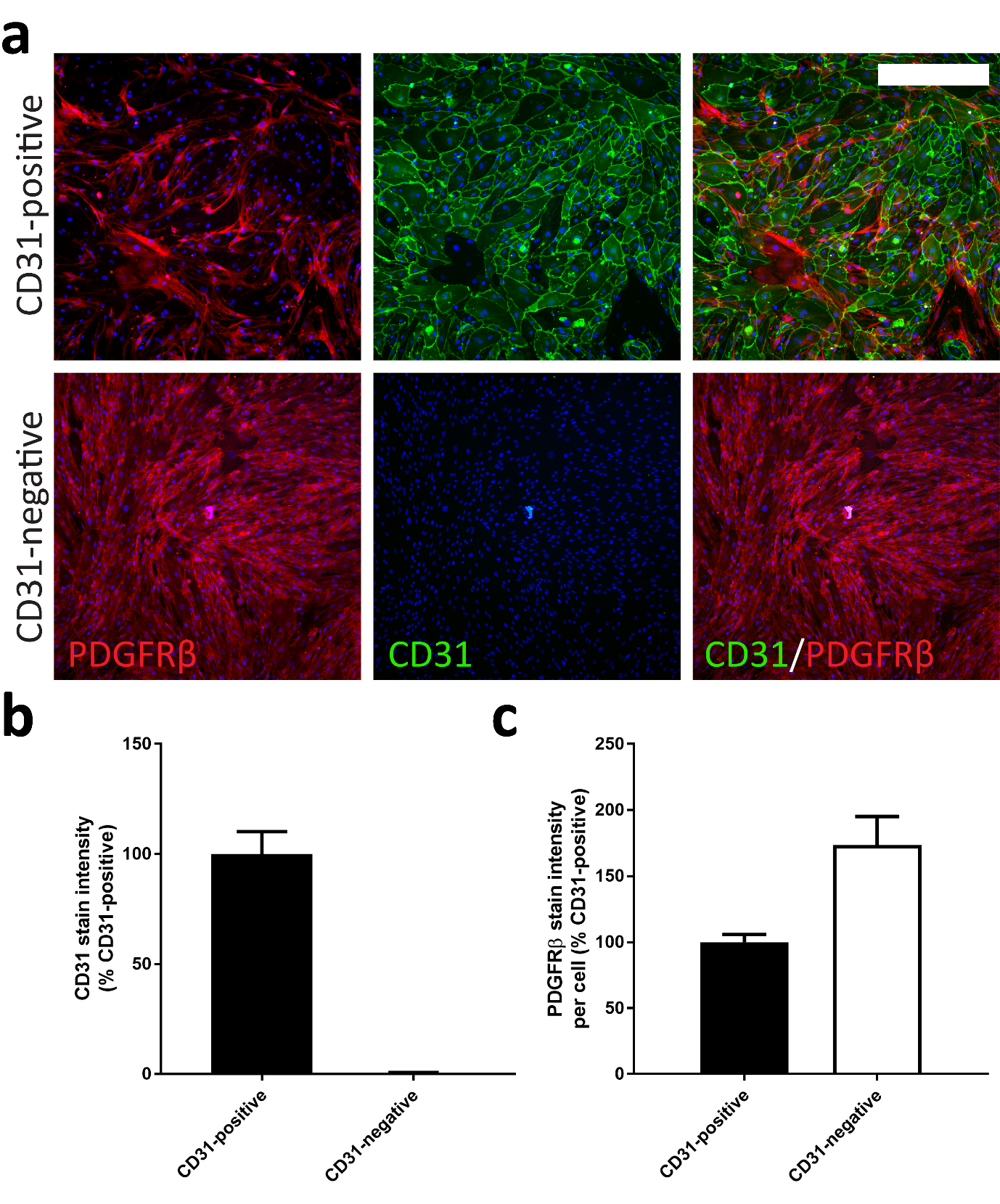


**Figure S1: Pericyte contamination develops in endothelial cultures in the absence of puromycin selection following cell sorting.** Endothelia were grown as described above, but selection of endothelia by CD31 magnetic activated cell sorting (MACS) performed at P1. The CD31-positive fraction was grown in a 96 well plate, fixed and immunostained. a) Representative images and b) approximation of the purity of endothelia in the CD31-positive fraction of MACS-sorted cultures. Scale bar = 500 µm. Mean ± SEM, n = 3 technical replicates.


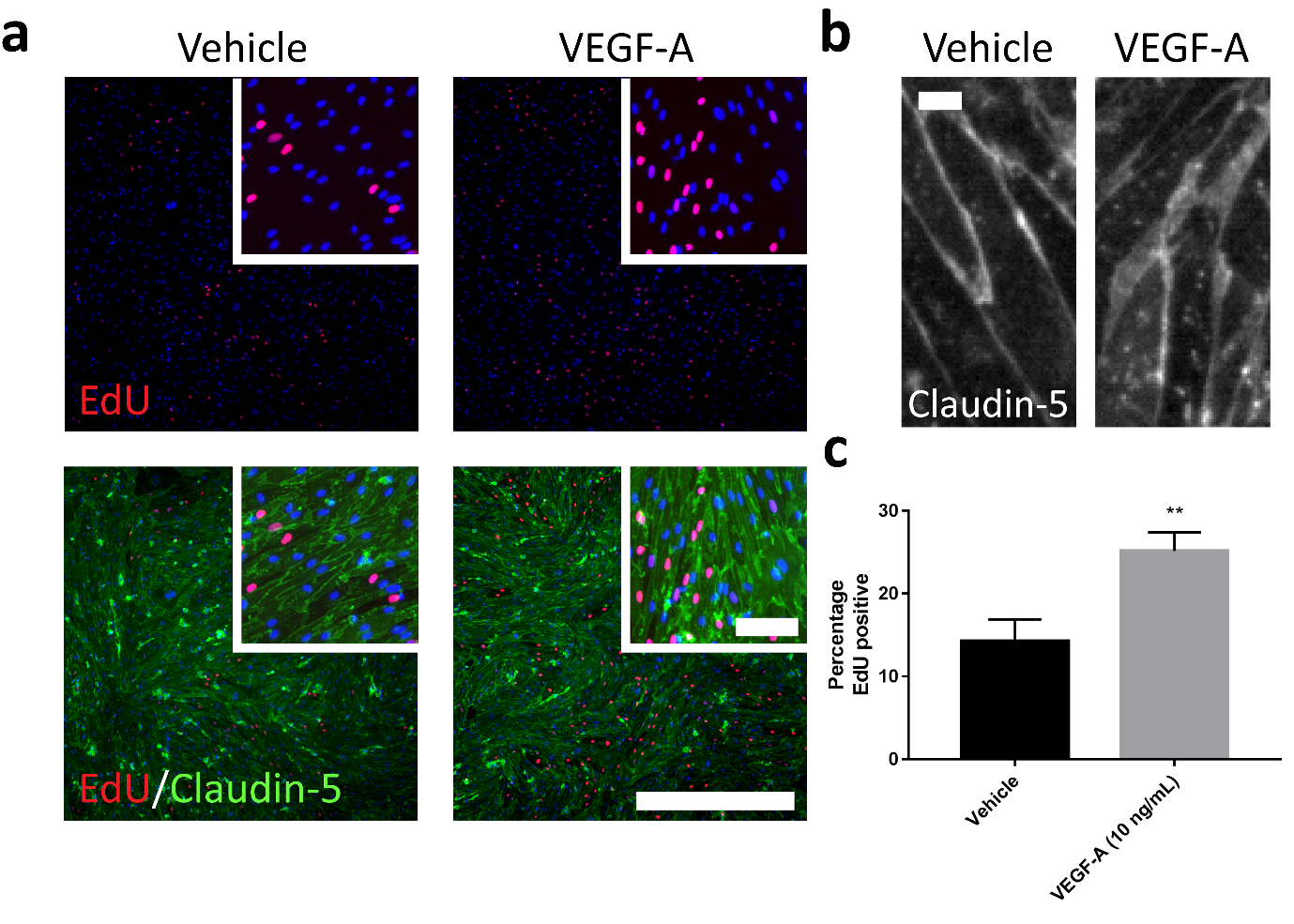


**Figure S2: Brain endothelial cultures are proliferative and have a proliferative response to VEGF-A.** Primary brain endothelial cultures were stimulated with VEGF-A (10 ng/mL) or vehicle (0.1% BSA in PBS), and treated with EdU before being allowed to proliferate for 48 hours. a) Representative immunostaining of endothelia for claudin-5 and EdU. b) High magnification images of claudin-5 immunostaining. Scale bar = 10 µm. c) Quantification of EdU-positive endothelia (n = 2, mean ± SEM).


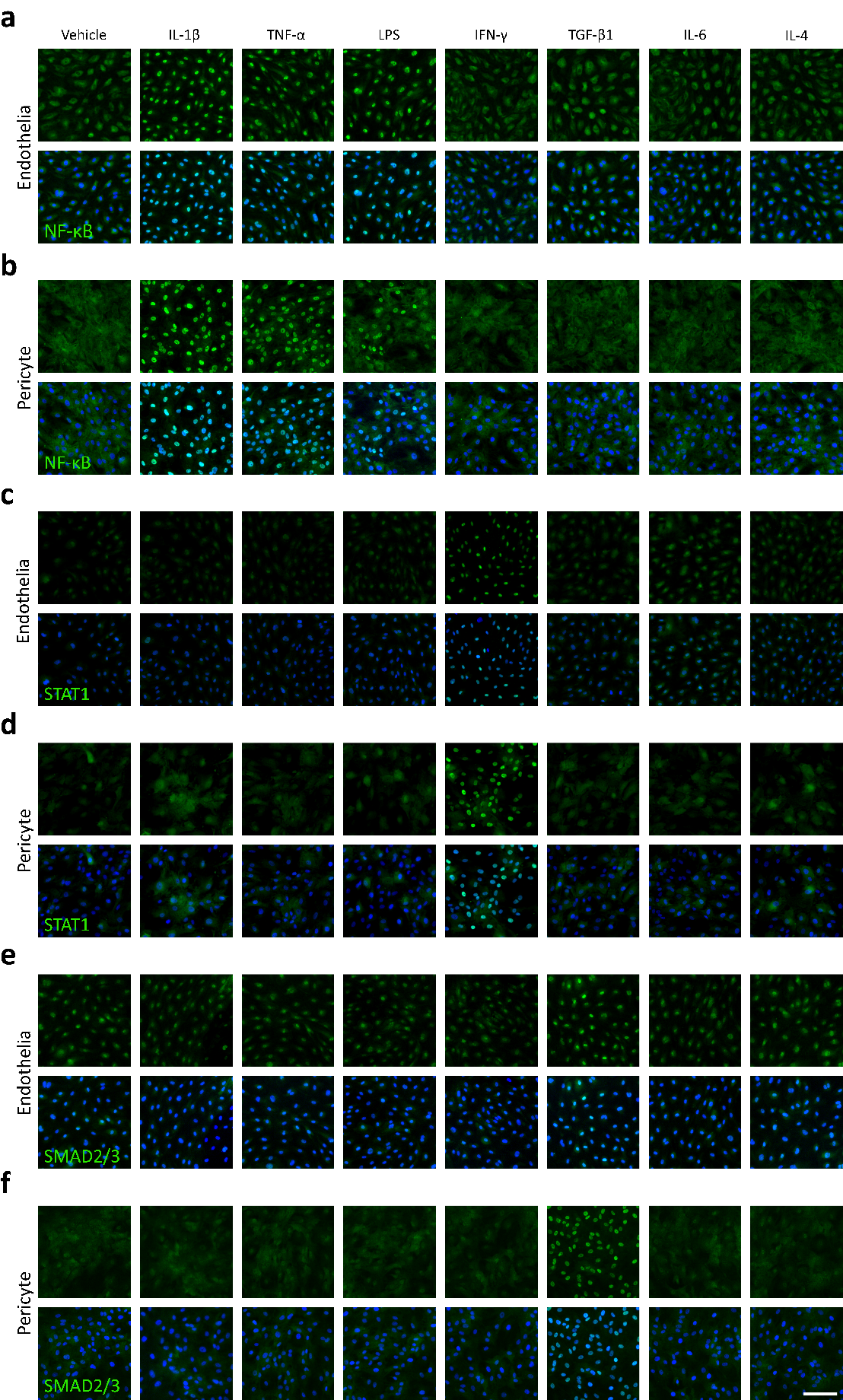


**Figure S3: Representative images showing the pathways activated by inflammatory stimuli in pericytes and endothelial cells, quantified in figure 3.** NFκB activation in response to the inflammatory panel in a) endothelia and b) pericytes. STAT1 activation in in response to the inflammatory panel in c) endothelia and d) pericytes. SMAD2/3 activation in response to the inflammatory panel in e) endothelia and f) pericytes. Scale bar = 200 µm.


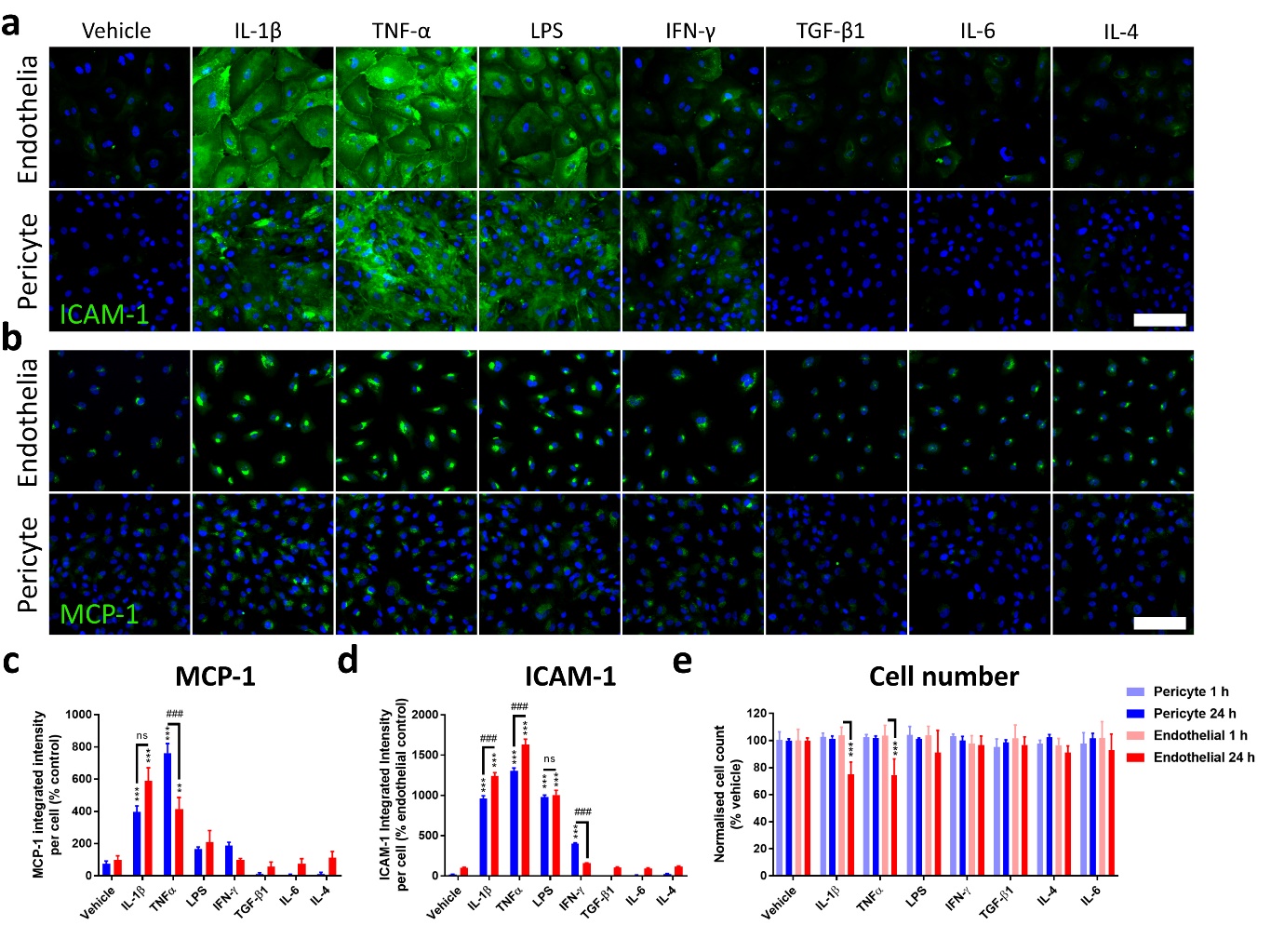


**Figure S4: Inflammatory response of endothelia and pericytes to the inflammatory stimulus panel analysed by immunocytochemistry.** Endothelia or pericytes were treated with inflammatory stimuli (10 ng/mL, 24 h) and fixed, before immunostaining. Representative images of a) ICAM-1 and b) MCP-1 immunostaining following treatment with inflammatory stimuli. Scale bar = 200 µm. Quantification of immunostaining of c) MCP-1 and d) ICAM-1 immunostaining following treatment with inflammatory stimuli. e) Normalised cell count of endothelia and pericytes treated with inflammatory stimuli. n = 3, mean ± SEM. Scale bar = 200 µm.

**
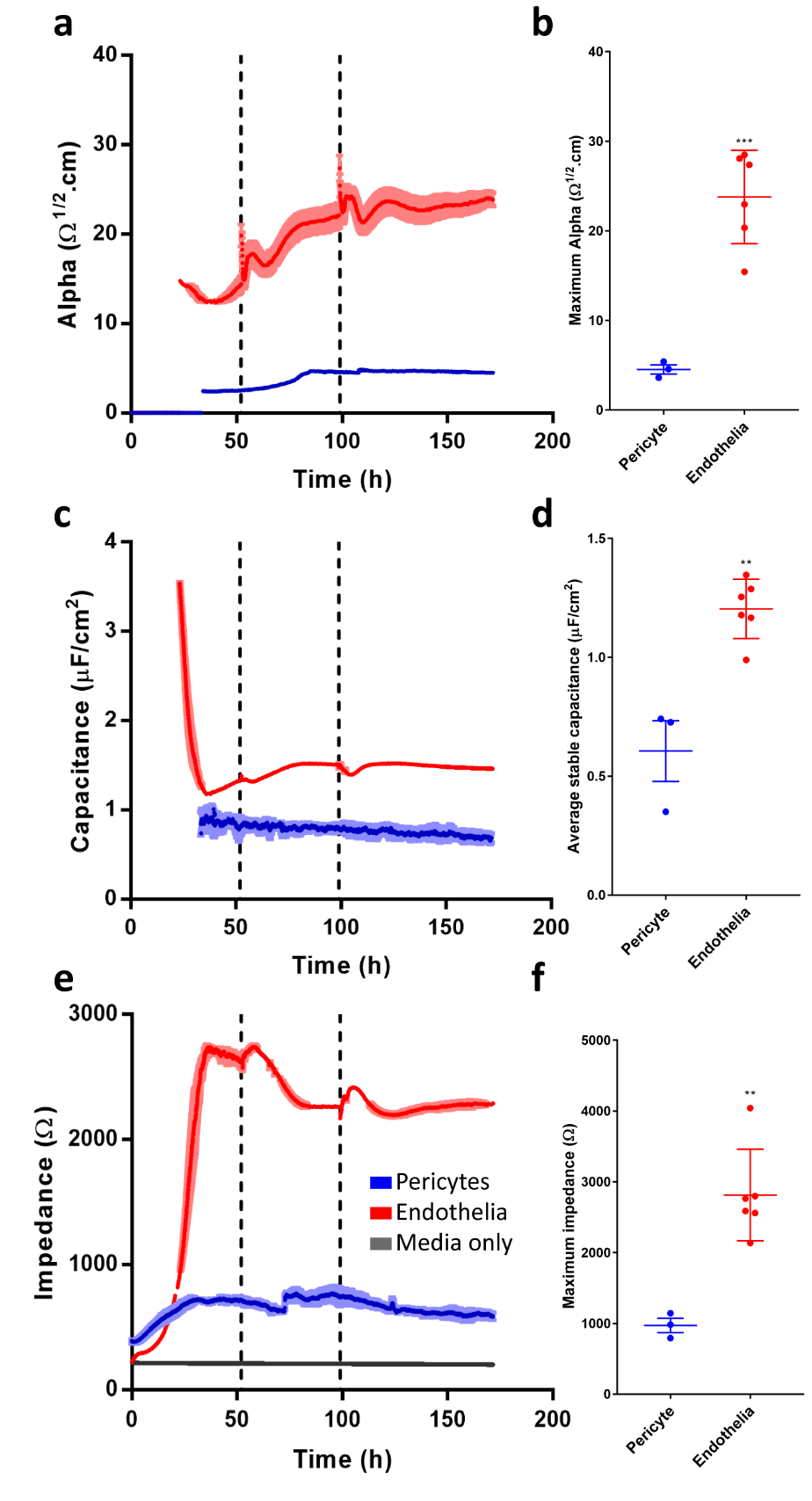
**

**Figure S5: Brain endothelia generate robust barrier properties in vitro.** Continuous measurement of barrier properties of endothelia and pericytes was performed using ECIS for up to eight days, and alpha, capacitance, and raw impedance measures recorded. a) Representative alpha trace from primary endothelia and pericytes. b) Maximal alpha measurement in endothelial cells and pericytes c) Representative capacitance trace from primary endothelia and pericytes. d) Average stable capacitance measurement in endothelial cells and pericytes. e) Representative raw impedance trace from primary endothelia, pericytes and a media-containing, cell-free well. Dashed lines denote when media was changed. f) Maximal impedance measurement in endothelial cells and pericytes. Dashed lines denote the point the media was changed. Timecourse data are presented as mean ± SEM from duplicate wells. Pooled data are presented as mean ± SEM from six independent cases.
